# Chromium pollution and its effects on soil health, microbial diversity, and sugarcane production in the Vellore sugarcane belt: a metagenomic and geochemical assessment

**DOI:** 10.1007/s11274-026-05402-1

**Published:** 2026-11-13

**Authors:** S. Gowtham, M. P. Sugumaran, Ettiyagounder Parameswari, A. R. U. Pragadeesh, T. Kalaiselvi, N. A. Saravanan, M. Elayarajan, M. Selva Kumar, K. Surya

**Affiliations:** 1 Department of Environmental science, Tamil Nadu Agricultural University, Coimbatore, Tamil Nadu India; 2 Hawkesbury Institute for the Environment, Western Sydney University, Penrith, NSW Australia; 3 Department of Agricultural Microbiology, Tamil Nadu Agricultural University, Coimbatore, Tamil Nadu India; 4 Sugarcane Research Station (SRS), Tamil Nadu Agricultural University, Vellore, Tamil Nadu India; 5 Department of Soil science and agriculture chemistry, Tamil Nadu Agricultural University, Coimbatore, Tamil Nadu India; 6 Agro Climate Research Centre, Tamil Nadu Agricultural University, Coimbatore, Tamil Nadu India; 7 Department of Agronomy, Tamil Nadu Agricultural University, Coimbatore, Tamil Nadu India

**Keywords:** Chromium speciation, Sugarcane rhizosphere, Nanopore 16S rRNA sequencing, Soil bacterial diversity, Tannery effluent, Chromate resistance genes

## Abstract

Disposal of tannery effluent has elevated soil chromium (Cr) across the Vellore sugarcane belt of southern India, yet its integrated consequences for soil health, bacterial communities and sugarcane productivity remain poorly characterised. Nine composite soil samples from a recognised tannery-affected belt were pre-stratified into High, Low and Near-threshold Cr sites. Soils and irrigation water were geochemically characterised for total and speciated heavy metals and profiled by full length (V1-V9) 16​S rRNA gene amplicon sequencing on an Oxford Nanopore GridION. Soil total Cr increased from 29.10 ± 0.14 to 180.51 ± 2.95 mg kg^− 1^, the alkaline-extractable Cr (VI) fraction rising from 2.92 ± 0.09 to 52.07 ± 0.11 mg kg⁻¹ and co-occurring Cd and Ni increasing across strata. Soil pH shifted alkaline (7.74 to 8.48), organic carbon declined by up to 31.7% and exchangeable Ca and Mg by up to 40.4%. Tissue Cr followed root > shoot > leaf but peaked at Near-threshold rather than High Cr sites, indicating bioavailability-driven uptake. Cane and stalk fresh weight declined 25.8–31.3% at High Cr sites, whereas sucrose, Brix, purity and fibre remained largely unchanged. Alpha diversity followed a threshold structured rather than linear dose response, with observed richness in High Cr levels reduced to 74–76% that in Low Cr levels alongside parallel declines in Shannon diversity and Pielou’s evenness. NMDS resolved three non-overlapping community clusters. A Cr-resilient core (*Sphingomonas panacis*, *Nitrospira lenta*) persisted alongside elevated prediction of *azoR/acpD* and *chrA* abundance, defining a chromium-tolerance signature in tannery-impacted sugarcane soils.

**Supplementary Information:**

The online version contains supplementary material available at https://doi.org/10.1007/s11274-026-05402-1.

## Introduction

Chromium (Cr) contamination of agricultural soils, largely driven by unregulated effluents from the leather tanning sector, has emerged as a critical threat to the ecological integrity of cropping systems (Xie et al. 2023). Recent global studies from approximately 28,000 georeferenced observations reveal that 14–17% of the world’s cropland is affected by toxic metal pollution. An estimated 0.9–1.4 billion people inhabit regions where metals and metalloids pose elevated public health and ecological risk (Hou et al. 2025). Among these elements, Cr occupies a distinctive position because of its dual oxidation chemistry, its strong coupling to industrial process waters, and its well documented ecotoxicity to plants and to soil microbiota (Hamilton et al. 2018; Zulfiqar et al. 2023; Srivastava et al. 2021).

In soils, Cr occurs predominantly in the relatively inert trivalent form (Cr^3+^), which is largely immobilised by precipitation, complexation and fixation on soil colloids (Hamilton et al. 2018; Zulfiqar et al. 2023). Anthropogenic inputs account for roughly 70% of total Cr accumulation in agricultural soils. The principal sources are tannery effluent, metal smelting, electroplating, pigment and paper manufacture, Cr-loaded fertilizers and biosolids, and long-term use of contaminated irrigation water (Elkelish et al. 2024; Zulfiqar et al. 2023; Srivastava et al. 2021). Hexavalent chromium (Cr^6+^) is the more hazardous species. It is a powerful oxidizing agent, a recognized respiratory carcinogen, and far more soluble, mobile and bioavailable than Cr^3+^. Its dominance is greatest under alkaline conditions, where the HCrO_4_^−^ and CrO_4_^2−^ oxyanions are electrostatically repelled by the negatively charged surfaces of clay minerals and humus and leach readily through the soil profile into groundwater (Hamilton et al. 2018; Gil-Cardeza et al. 2014; Srivastava et al. 2021; Zulfiqar et al. 2023).

India is among the countries most severely impacted by Cr pollution, with Kanpur, Ranipet, Vadodara and Talcher repeatedly identified as Cr contaminated hotspots (Singh et al. 2020; Zulfiqar et al. 2023; Karthikeyan et al. 2021; Rangasamy et al. 2015; Sinduja et al. 2022). The Vellore district of Tamil Nadu (12°00’-13°15’ N, 78°20’-79°50’ E), drained by the eastward flowing Palar River and its tributaries, is one of the largest leather tanning clusters in the country (Narayanan and Thirukumaran 2021; Kanchana et al. 2018; Pranavam et al. 2011; Kistan et al. 2017). More than 600 tanneries operate in the district, about 150 of them at Ranipet on the banks of the Palar, discharging approximately 3,122 kilolitres of Cr laden effluent per day (Kistan et al. 2017; Rangasamy et al. 2015; Pranavam et al. 2011). Disposal of tannery sludge and prolonged use of tannery impacted irrigation water have created chronic Cr contamination across the agricultural lands of Vellore and the adjoining taluks of Walajapet and Ranipet (Rangasamy et al. 2015; Karthikeyan et al. 2021; Sinduja et al. 2022). These contaminated lands are not marginal. They coincide with one of the most intensively cropped sugarcane tracts in Tamil Nadu, so that any impairment of soil function here is translated directly into the productivity of a commercially significant crop.

Tamil Nadu ranks first in India in sugarcane productivity, with an average cane yield of 101 t ha^− 1^ which is only half of the crop’s genetic potential of 203.7 t ha^− 1^, leaving a yield gap of roughly 50% (Karpagam et al. 2019). Bridging this gap requires an integrated understanding of the chemical, biological, and ecological constraints on cane cultivation, particularly in major cane growing regions such as the Vellore belt where extensive industrial Cr contamination coexists with intensive cropping (Zulfiqar et al. 2023; Karpagam et al. 2019; Karthikeyan et al. 2021). Survey-level studies have demonstrated that more than 90% of 65 soil samples collected across Vellore district contained chromium concentrations exceeding 200 mg kg^− 1^. This is well above the maximum permissible limits (100 mg kg^− 1^) prescribed by WHO/FAO (2007). Groundwater samples drawn from sites along the Ranipet, Walajapet and Vaniambadi corridor have been reported to contain Cr up to 38 mg L^− 1^, against the critical drinking water limit of 0.05 mg L^− 1^ (Karthikeyan et al. 2021; Rangasamy et al. 2015; Sinduja et al. 2022).

Soils contaminated to such Cr levels experience impairment across multiple, interrelated dimensions of soil health. Increasing Cr loading has been associated with statistically significant declines in soil pH, depletion of alkali-hydrolysable nitrogen, and disruption to the nitrogen and carbon cycles (Utobo and Tewari 2015; Zhang et al. 2021; Wyszkowska et al. 2001). Even Cr^3+^, which is less mobile than Cr, has been shown to inhibit dehydrogenases, urease, and acid/alkaline phosphatases at field relevant doses of 80–120 mg kg^− 1^ and to reduce the abundance of free-living nitrogen fixing *Azotobacter* spp. and actinomycetes in sandy loam soils (Utobo and Tewari 2015; Wyszkowska et al. 2001; Quazi and Aery 2014).

Soil microbial communities both drive and reflect these functional losses, and Cr contamination systematically reduces bacterial diversity and reshapes community structure (Zhu et al. 2023; Huang et al. 2021; Zhao et al. 2024). Along documented Cr pollution gradients, bulk soil bacterial abundance quantified by 16​S rRNA gene copy number has been observed to fall by almost four orders of magnitude from 9.75 to 5.97 log (copies) g^− 1^ dry weight as bulk-soil Cr concentration increases in China (Huang et al. 2021). Highly contaminated soils tend to host communities that are taxonomically narrower and less even, while tolerant lineages carrying chrA type chromate efflux genes become selectively enriched, indicating a pollution induced community tolerance response (Campillo-Cora et al. 2023; Zhao et al. 2024; Zhu et al. 2023). These compositions frequently correlate with suppressed carbon mineralization rates and reduced microbial biomass, directly compromising the nutrient cycling necessary for vigorous sugarcane development (Campillo-Cora et al. 2023). The consequences extend to the crop itself, because Cr competes with essential ions for uptake and translocation within the soil plant system. Cr enters root cells through phosphate and sulfate transporters as chromate and through Fe type carriers as Cr3+, causing antagonistic interactions with the uptake of Fe, Zn, P and S (Saleem et al. 2022; Srivastava et al. 2021; Sundaramoorthy et al. 2010; Singh et al. 2020). Cr stressed plants exhibit delayed germination, stunted growth, leaf chlorosis, photosynthetic impairment, oxidative damage and substantial yield reductions across cereals, legumes, oilseeds, vegetables and fibre crops (Mathur et al. 2016; Elkelish et al. 2024; Saud et al. 2022; Zulfiqar et al. 2023; Saleem et al. 2022).

Geochemical characterization is the measurement of total Cr with hexavalent Cr and trivalent Cr fractions in soil, irrigation water, and plant tissues, it provides quantitative insight into Cr speciation, mobility, bioavailability, and long-term persistence in agricultural soils (Filgueiras et al. 2002; Nannoni and Protano 2016; Rauret et al. 1999; Zulfiqar et al. 2023; Hamilton et al. 2018; Gil-Cardeza et al. 2014). Culture independent 16S rRNA profiling of total environmental DNA resolves bacterial community structure and predicted functional gene repertoire across metal stress gradients at fine resolution (Singh et al. 2022; Feng et al. 2018; Pei et al. 2018; Pradhan et al. 2022; Huang et al. 2021).

Previous work in the Vellore, Ranipet and Palar belt has been exclusively geochemical, comprising Cr speciation and contamination mapping in soil and sludge (Rangasamy et al. 2015; Kistan et al. 2017; Sinduja et al. 2022) and groundwater quality assessment (Pranavam et al. 2011; Kanchana et al. 2018; Sinduja et al. 2023). None of these studies characterised the soil microbial community. This is the first study from Vellore and adjoining areas to profile the soil bacterial community of Cr contaminated agricultural soil using full length (V1-V9) 16​S rRNA nanopore sequencing, and to couple that community data with Cr speciation and agronomic performance of sugarcane in the same fields.

This study provides an integrated geochemical and microbiological assessment of Cr pollution and its consequences for soil health, microbial diversity and sugarcane production in the Vellore sugarcane belt. The specific objectives were: (i) to quantify Cr accumulation and geochemical partitioning across representative sites of the belt, alongside key soil physico chemical indices (pH, electrical conductivity, organic carbon, available NPK); (ii) to characterise the soil bacterial community and its predicted functional potential along the resulting contamination gradient; and (iii) to relate both to sugarcane growth and yield in field grown cane from the same sites, so that the geochemical and microbiological findings can be linked directly to agronomic performance.

## Materials and methods

### Study site

The study was conducted in the sugarcane growing belt of the Vellore, Ranipet and Tirupattur administrative divisions of Tamil Nadu, southern India. The region lies within the Palar river basin, has an arid to semi-arid climate, and supports a dense cluster of leather tanning units whose effluents and solid wastes constitute the principal anthropogenic source of chromium in the surrounding agricultural land (Rangasamy et al. 2015; Sinduja et al. 2022). Groundwater is the dominant source of irrigation for field crops in this region (Sinduja et al. 2023).

**Fig. 1:**
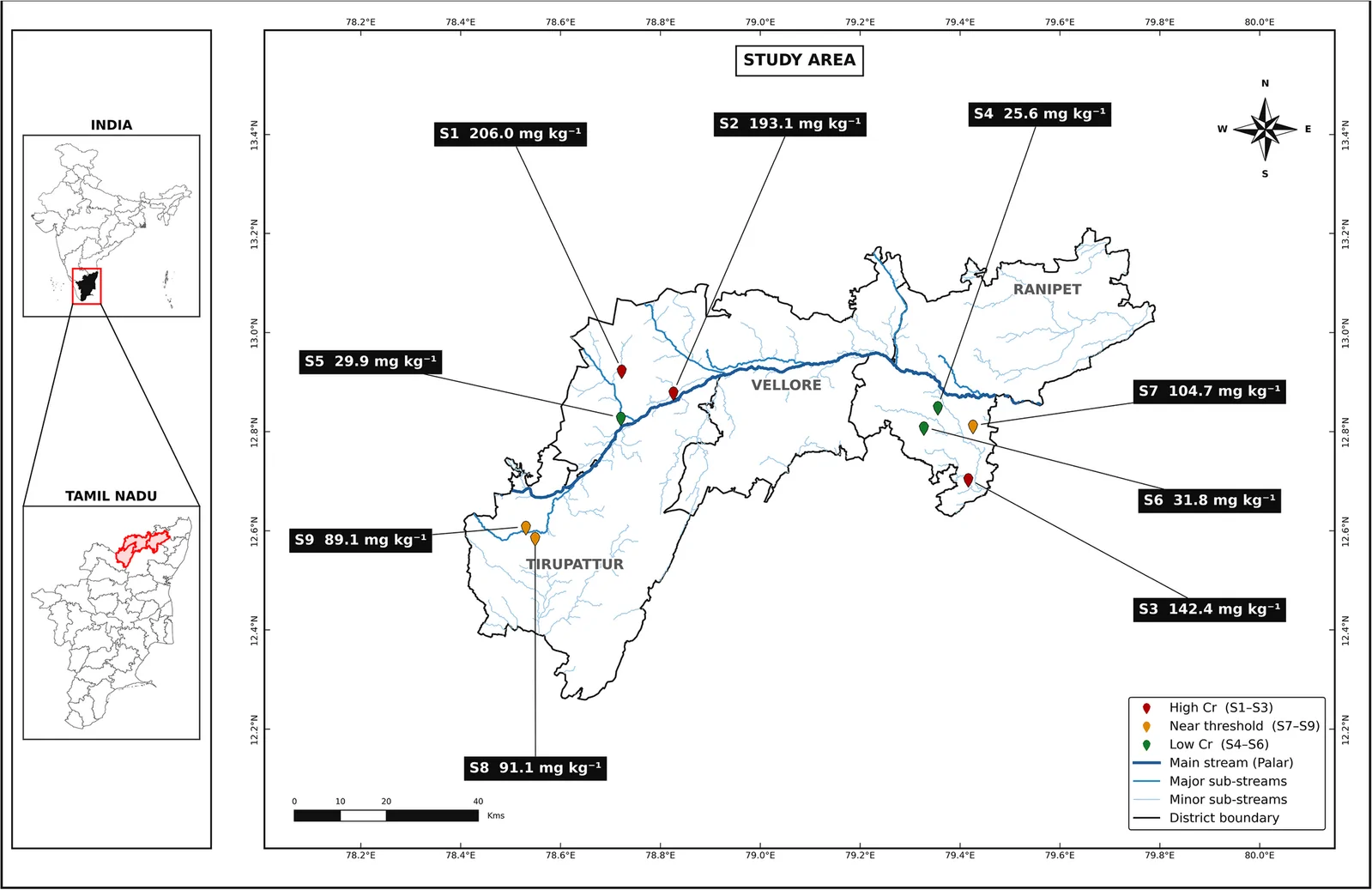
Location of the nine soil sampling sites (S1-S9) across the Vellore, Ranipet and Tirupattur administrative divisions of Tamil Nadu, India, with soil total chromium concentration (mg kg^− 1^) labelled at each point. Sites are colour coded by chromium exposure class: red = high (S1-S3), yellow = near threshold (S7-S9) and green = low (S4-S6)

Nine sugarcane fields were selected following a reconnaissance survey and were stratified into three chromium contamination classes based on soil total Chromium: High Cr, near threshold Cr and Low Cr, each represented by three independent fields (Kistan et al. 2017; Karthikeyan et al. 2021; Rangasamy et al. 2015; Sinduja et al. 2022; Hou et al. 2025). High Cr class comprised Peranbuttu (S1), Valathur 2 (S2) and Pennagar (S3); Low Cr class comprised Ladavaram (S4), Aiyathambattu (S5) and Anathangal (S6); and the near-threshold class comprised Nethapaakam (S7), Athurkuppam (S8) and Athithangal (S9). Fields were stratified on the basis of soil total Cr measured during the reconnaissance survey and benchmarked against the maximum permissible limit of 100 mg kg^− 1^ for agricultural soils (WHO/FAO 2007). Fields containing less than half the permissible limit (< 50 mg kg^− 1^) were assigned to the Low Cr class; fields within ± 25% of the limit (75–125 mg kg^− 1^) to the Near-threshold class; and fields exceeding 1.4 times the limit (> 140 mg kg^− 1^) to the High Cr class. The nine fields separated cleanly into these three groups, spanning 25.6–31.8 mg kg^− 1^ (Low Cr), 89.1-104.7 mg kg^− 1^ (Near-threshold) and 142.4–206.0 mg kg^− 1^ (High Cr), with no site falling between 31.8 and 89.1 mg kg^− 1^ or between 104.7 and 142.4 mg kg^− 1^. Class assignment was confirmed by the full analytical determinations reported in Table 3. Sampling at each field was replicated four times to provide an estimate of within site variance (Ramsey et al. 1995; Ramsey 2015). The geographic coordinates of the nine sites are given in Table 1, and their spatial distribution together with the measured soil total Cr concentrations is shown in Fig. 1.

**Table 1:** Chromium contamination class, site code and geographic coordinates of the nine sampling sites

Contamination class	Site (sample code)	Latitude (° *N*)	Longitude (° E)	Soil total Cr (mg kg^− 1^)
High Cr	Peranbuttu (S1)	12.9238	78.7226	206.0
High Cr	Valathur 2 (S2)	12.8793	78.8263	193.1
High Cr	Pennagar (S3)	12.7046	79.4167	142.4
Low Cr	Ladavaram (S4)	12.8499	79.3556	25.6
Low Cr	Aiyathambattu (S5)	12.8284	78.7209	29.9
Low Cr	Anathangal (S6)	12.8091	79.3276	31.8
Near threshold	Nethapaakam (S7)	12.8124	79.4259	104.7
Near threshold	Athurkuppam (S8)	12.5861	78.5492	91.1
Near threshold	Athithangal (S9)	12.6083	78.5306	89.1

### Soil, water and plant sampling

At each of the nine sites, rhizosphere soil, the irrigation water source serving that field and sugarcane leaf, shoot and root tissue were collected concurrently from the same plants, so that soil, water and plant data are directly comparable within a site (Sundaramoorthy et al. 2010; Mathur et al. 2016; Saleem et al. 2022; Zulfiqar et al. 2023). Four field replicates were collected per site, giving 36 observations for each matrix. The geographic coordinates of every sampling point were recorded in the field (Sinduja et al. 2022).

Soil samples were ground and passed through a sieve, and the fraction < 150 μm was retained for acid digestion and metal determination. Plant material was washed, oven dried at 60–65 °C to constant weight and ground to pass a 1 mm sieve before digestion (Pandey et al. 2016; Saleem et al. 2022). Irrigation water samples were collected from the source used at each field and analysed following standard procedures (APHA 2012).

### Analytical methods

All soil, water and plant determinations followed standard analytical procedures; the method and reference standard adopted for each parameter are summarised in Table 2. Soil pH and electrical conductivity were determined in a 1:2.5 (w/v) suspension and a saturation paste extract respectively (Jackson 1973), organic carbon by Walkley-Black wet oxidation (Walkley and Black 1934), and available N, P, K and S and exchangeable Ca, Mg and Na as detailed in Table 2 (Olsen et al. 1954; Subbiah and Asija 1956; Williams and Steinbergs 1959; Jackson 1973). Irrigation water was characterised for pH, EC, hardness, alkalinity, carbonate, bicarbonate, chloride, sulphate, nitrate and major cations following standard procedures (Jackson 1973; APHA 2012; Kanchana et al. 2018; Sinduja et al. 2023).

**Table 2:** Analytical methods and reference standards adopted for the physico-chemical and elemental characterisation of soil, irrigation water and sugarcane tissue

Parameter	Matrix	Method	Reference
pH	Soil, water	pH meter with combined glass electrode (1:2.5 soil: water suspension for soil)	Jackson (1973)
EC (dS m^− 1^)	Soil, water	Conductivity meter (saturation paste extract for soil)	Jackson (1973)
Organic carbon (%)	Soil	Chromic acid wet oxidation; organic matter = organic carbon × 1.724	Walkley and Black (1934)
Available nitrogen (kg ha^− 1^)	Soil	Alkaline KMnO_4_ distillation	Subbiah and Asija (1956)
Available phosphorus (kg ha^− 1^)	Soil	0.5 M NaHCO_3_ (pH 8.5) extraction; ascorbic acid molybdenum-blue colorimetry at 882 nm	Olsen et al. (1954)
Available potassium (kg ha^− 1^)	Soil	Neutral normal NH_4_OAc extraction - flame photometry	Jackson (1973)
Exchangeable Ca and Mg (cmol(p^+^) kg^− 1^)	Soil	Neutral normal NH_4_OAc extraction - versenate (EDTA) titration	Jackson (1973)
Exchangeable Na (cmol(p^+^) kg^− 1^)	Soil	Neutral normal NH_4_OAc extraction - flame photometry	Jackson (1973)
Available sulphur (mg kg^− 1^)	Soil	0.01 M Ca(H_2_PO_4_)_2_ extraction -turbidimetric BaSO_4_ method at 420 nm	Williams and Steinbergs (1959)
Total hardness and total alkalinity (mg L^− 1^)	Water	EDTA complexometric titration; H_2_SO_4_ titration	APHA (2012)
Chloride (mg L^− 1^)	Water	Mohr’s argentometric titration	APHA (2012)
Sulphate (mg L^− 1^)	Water	Turbidimetric method	APHA (2012)
Nitrate (mg L^− 1^)	Water	UV spectrophotometric method	APHA (2012)
Carbonate and bicarbonate (mg L^− 1^)	Water	Titrimetric method	APHA (2012)
Calcium and magnesium (mg L^− 1^)	Water	Versenate (EDTA) titration	Jackson (1973)
Sodium and potassium (mg L^− 1^)	Water	Flame photometry	Jackson (1973)
Total Cr, Cd, Ni and Pb (mg kg^− 1^)	Soil	HNO_3_:HCl (1:3, v/v) microwave-assisted digestion (soil, USEPA 3050B) - MP-AES (Agilent 4210)	USEPA (1996)
Hexavalent chromium (mg kg^− 1^)	Soil	Alkaline Na_2_CO_3_/NaOH digestion (soil, USEPA 3060 A) − 1,5-diphenylcarbazide colorimetry at 540 nm	USEPA (1992)
Total chromium (mg kg^− 1^ dry weight)	Plant (root, shoot, leaf)	Oven-dried, ground tissue; HNO_3_:HClO_4_ (4:1, v/v) di-acid digestion - MP-AES (Agilent 4210)	Allen (1974)

Soil subsamples (0.5–1.5 g, < 150 μm) were microwave-digested in HNO_3_:HCl (1:3, v/v) following USEPA Method 3050B, and water samples following USEPA Method 3005 A (Moor et al. 2001; Gaudino et al. 2007; Ahmad et al. 2021). Digests were analysed for Cr, Cd, Ni, Pb, Fe, Zn, Mn and Cu by microwave plasma atomic emission spectrometry (MP-AES; Agilent 4210, Agilent Technologies, USA), with operating conditions set according to the multi-metal workflow of Ahmad et al. (2021) (Nannoni and Protano 2016; Ranieri et al. 2021). Hexavalent Cr was extracted from soil by alkaline digestion with Na_2_CO_3_/NaOH at pH > 11 (USEPA Method 3060 A) and quantified spectrophotometrically at 540 nm by the 1,5-diphenylcarbazide method (USEPA Method 7196 A; Filgueiras et al. 2002; Hamilton et al. 2018, 2020); water samples were analysed directly. Trivalent Cr was obtained by difference, Cr(III) = total Cr - Cr(VI) (Gil-Cardeza et al. 2014; Zulfiqar et al. 2023; Sinduja et al. 2022). Oven-dried leaf, shoot and root tissue was digested in HNO_3_:HClO_4_ (4:1, v/v) and analysed for Cr under the same MP-AES conditions (Pandey et al. 2016; Saleem et al. 2022). Every digestion batch included a reagent blank and a certified reference material (NIST SRM 2701 for soil; a plant certified reference material for tissue), and calibration was verified daily (Pueyo et al. 2001; Rauret et al. 1999; Allen 1974).

Contamination status was expressed on the Cr-specific axes of total Cr, Cr(VI) and Cr(III) rather than through a generic multi-metal pollution index, with Cd, Ni and Pb retained as co-occurring analytes providing geochemical context (Kistan et al. 2017; Karthikeyan et al. 2021; Sinduja et al. 2022).

Vegetative growth, stalk morphometric, cane yield and juice-quality parameters were recorded as described in Supplementary Methods S1 (Vinicius et al. 2017; Shukla et al. 2020; Saranraj et al. 2023; Karpagam et al. 2019; Meena et al. 2020). Full analytical protocols, instrument conditions and quality-control details are provided in Supplementary Methods S1.

### Soil metagenomic profiling (16​S rRNA) and bioinformatics pipeline

Total metagenomic DNA was extracted from soil samples (9 sites) using the Qiagen DNeasy PowerSoil Pro Kit as per the guidelines (Demkina et al. 2023; Jensen et al. 2024). Isolated metagenomic DNA was stored at − 20 °C for further research (Smenderovac et al. 2024). DNA obtained was evaluated for purity and concentration using a NanoDrop spectrophotometer, and DNA purity was checked using agarose gel electrophoresis on a 0.8% agarose gel before proceeding with PCR (Johnston et al. 2016; Schröttner et al. 2016). NanoDrop measurements, particularly the A260/A280 ratios, which ranged from about 1.8 to 2.0, were used as indicators of DNA quality (Demkina et al. 2023; Jensen et al. 2024; Smenderovac et al. 2024). A nanopore library was prepared using samples with optimal purity and concentration (Stevens et al. 2023). Metagenomic DNA (50 ng) from each sample was used for PCR amplification using the 16​S barcoding kit, which includes 16​S rRNA gene (V1-V9 region) V15’AGAGTTTGATCMTGGCTCAG 3’V95’TACGGYTACCTTGTTACGACTT 3’, Oxford Nanopore Technologies, Oxford, UK) (Boutsika et al. 2024; Matsuo et al. 2021). The expected amplicon size was approximately 1500 bp (Chandna et al. 2013; Ihsan et al. 2020). Amplification was confirmed by agarose gel electrophoresis (1% agarose gel) with ethidium bromide (Chandna et al. 2013; Qian et al. 2001). The amplicons were cleaned using 1.6× AMPure XP magnetic beads according to the instructions to remove primer dimers and residual reaction contaminants (Boutsika et al. 2024; Kipp et al. 2023).

### Preparation of the library and barcoding

From each sample, 50 ng of purified PCR product was subjected to end repair using the NEBNext^®^ Ultra II End Repair Kit, followed by cleanup with 1× AMPure XP beads (​Kipp et al. 2023; Lee et al. 2026). Barcoding adapters were then ligated using NEB Blunt/TA Ligase, and the ligation products were purified again with 1× AMPure XP beads (Karunarathna et al. 2025; Kipp et al. 2023; Phelan et al. 2019). The adapter ligated fragments were quantified using a Qubit fluorometer and subsequently amplified with LongAmp^®^ Taq 2× Master Mix to incorporate unique sample barcodes (Boutsika et al. 2024; Karunarathna et al. 2025; Kipp et al. 2023). The barcoded amplicons were purified using 1.6× AMPure XP beads (Lee et al. 2026). After quantification, all barcoded samples were pooled in equimolar amounts and subjected to a second end repair reaction using the NEBNext^®^ Ultra II End Repair Kit (Boutsika et al. 2024; Kipp et al. 2023; Lee et al. 2026). The final sequencing library was purified with AMPure XP beads and was eluted in 15 µL of elution buffer for nanopore sequencing (Karunarathna et al. 2025; Phelan et al. 2019).

### Sequencing and data processing

Nanopore sequencing was carried out on the GridION platform (Boutsika et al. 2024; Steinig and Coin 2022). Raw signal files were initially base-called and demultiplexed using Guppy v6.0.6, a highly accurate model that also performed barcode demultiplexing to obtain sample specific FASTQ files (Langsiri et al. 2023; Murigneux et al. 2021; Santos et al. 2020). To obtain quality filtered reads, FAST5 files were additionally processed with Guppy v2.3.4, producing FASTQ pass reads with a mean Q-score > 7 and ensuring retention of reliable sequences for downstream taxonomic and diversity analyses (Murigneux et al. 2021; Sanderson et al. 2018). Adapter and barcode trimming was performed using Porechop v0.2.4 (https://github.com/rrwick/Porechop) with default parameters (10,000 check reads, an adapter threshold of 90 and an end size of 150), enabling detection of ligation junctions and removal of internal barcodes (Murigneux et al. 2021; Sanderson et al. 2018; Santos et al. 2020). Subsequent quality filtering was conducted with NanoFilt v2.8.0, retaining reads with Q ≥ 10 (Santos et al. 2020; Steinig and Coin 2022). The final set of high quality, demultiplexed FASTQ reads was used for microbial community characterization, encompassing taxonomic classification, diversity assessment and functional analysis (Kurokochi et al. 2021; Santos et al. 2020; Veselovsky et al. 2025).

### Bioinformatics, taxonomic classification and diversity analyses

Quality-controlled, length filtered reads obtained from each sample were processed using MetONTIIME v1.3.2 (Ferrato et al. 2021) with parameters optimized for nanopore data (sequence type nanopore, minimum length 1000, maximum length 1800, minimum quality 10). Amplicon Sequence Variants (ASVs) were inferred following error correction and sequence denoising procedures, allowing discrimination of biological sequences at single-nucleotide resolution rather than clustering sequences into operational taxonomic units.Relative abundances were calculated by normalizing read counts per taxon (Weiss et al. 2017), and taxonomic profiles were tested across samples. ASV feature table and metadata were further analyzed using a SILVA database. Feature tables were also collapsed to genus level and to species level (“level-7”) using QIIME2’s taxa collapse function for taxon-resolved downstream analysis and exported as tab-separated abundance tables for import into R (v4.3.3). All downstream ecological and statistical analyses were performed in R (v4.3.3) using the vegan package, with supporting packages MASS and ggplot2 for visualization. Feature tables were analyzed at the species level.

Alpha diversity indices, which include Chao1 richness, Pielou’s evenness, and Shannon and Simpson diversity, were computed using the vegan package v2.6-4 (Oksanen et al. 2022). Group differences were tested using two sided Wilcoxon rank sum tests and Welch’s t-tests. Beta diversity across the samples was assessed from Bray Curtis dissimilarity matrices computed on the rarefied species level abundance table and visualized via non-metric multidimensional scaling using the metaMDS function in the vegan package (v2.6-4) in R (v4.3.3), with the following settings: distance = “bray”, k = 2 dimensions, autotransform = FALSE, trymin = 20 random starts, trymax = 200, model = “global” non-metric regression, and a maximum of 1,000 iterations per run until two consecutive runs converged to the same configuration with a stress value below 0.20, indicating an acceptable two dimensional representation of the rank order dissimilarity structure (Huang et al. 2021; Dai et al. 2023). Differences in community composition among the three chromium contamination strata were tested on the same Bray Curtis dissimilarity matrices by permutational multivariate analysis of variance implemented in vegan via adonis2 with 999 permutations and method = “bray”, with pairwise PERMANOVA between stratum pairs performed where the overall F-test was significant (*p* < 0.05). Microbial co-occurrence networks were constructed from Spearman rank correlations among the most abundant genera (Hmisc::rcorr), with edges retained where |ρ| > 0.6 and *p* < 0.05. Networks were visualized and characterized (node degree, betweenness, closeness, local clustering coefficient, global transitivity, density) using the igraph package.

To infer functional metagenomic potential from 16​S rRNA amplicon data, PICRUSt2 (v2.5.2) was employed. Briefly, amplicon sequence variants (ASVs) were phylogenetically placed into a reference tree using EPA-NG and GAPPA, after which hidden-state prediction (HSP) was applied to estimate KEGG Ortholog (KO) abundances for each sample. In addition to KO prediction, Clusters of Orthologous Groups (COG) functional categories were simultaneously predicted through the PICRUSt2 pipeline, providing a complementary annotation framework for assessing the functional repertoire of the microbial community. Predicted KO and COG profiles were normalized by 16​S rRNA gene copy number to account for variation across taxa. KO abundances were subsequently aggregated into KEGG pathway categories, while COG abundances were summarized into broader COG functional categories, enabling a comprehensive ecological interpretation of community-level functional potential.

### Statistical analysis

One-way analysis of variance was performed for each measured variable, and where the overall F-test was significant (*p* < 0.05), pairwise comparisons among treatment means were carried out using Tukey’s honestly significant difference post hoc test at α = 0.05 (Wakshum and Sharma 2018; Meena et al. 2020; Shukla et al. 2020; Farooqi et al. 2024). Bivariate relationships among soil, water and plant-tissue chromium and agronomic variables were characterized by Pearson correlation coefficients (*r*) with two-tailed *p*-values, supported by Benjamini-Hochberg false discovery rate correction (*q* < 0.05) for multiple comparisons (Gołębiewski et al. 2014; Yin et al. 2015; Hu et al. 2021; Dai et al. 2023; Li et al. 2024). Principal component analysis of standardized (z-scored) soil, water, plant tissue and agronomic variables was used to visualize the overall similarity among treatments across the multi parameter space (Hu et al. 2021; Dai et al. 2023; Li et al. 2024). All statistical analyses were performed in Python (SciPy ≥ 1.10 and statsmodels ≥ 0.14).

## Results

### Soil physicochemical properties and chromium contamination gradient

Soil samples collected across the Vellore sugarcane belt were classified into three chromium-contamination categories: Low, Near-threshold, and High based on their total chromium loading, and were compared for physicochemical and heavy metal parameters (Table 3). Soil pH increased with contamination level (9.6%), from 7.74 ± 0.13 in uncontaminated soils (low threshold limit) to 8.48 ± 0.02 in Highly contaminated soils (*p* < 0.001), indicating a shift toward alkalinity in chromium affected soils. Electrical conductivity (EC) rose sharply from 0.20 ± 0.00 to 0.43 ± 0.01 dS m⁻¹ at Near-threshold sites (+ 116.9%, *p* < 0.001) before partially declining at Highly contaminated sites (+ 66.1% relative to Low), suggesting non-monotonic salinisation associated with chromium loading.

Soil fertility indicators were consistently depressed under elevated chromium fractions. Organic carbon declined by 31.7% at Near-threshold and 20.0% at Highly contaminated sites (*p* < 0.001), while exchangeable calcium and magnesium fell steadily across the gradient, reaching reductions of 29.2% and 40.4% respectively at Highly contaminated sites (both *p* < 0.001). Available phosphorus, potassium, sulfur and exchangeable sodium all showed a non-monotonic pattern, reduction at Near-threshold sites (− 45.6%, − 34.7%, − 38.7% and − 26.2%, respectively) followed by partial or full recovery at Highly contaminated sites (all *p* < 0.001). This pattern reflects how chromium disrupts the availability of nutrients in the soil. At the highest levels of contamination, however, the effect seems to ease slightly may be due to the changes in redox conditions and sorption processes alter how chromium interacts with the soil.

All measured heavy metals increased sharply with the contamination gradient (Table 3). Total chromium rose from 29.10 ± 0.14 mg kg^− 1^ (Low) to 94.97 ± 1.23 (Near, + 226.3%) and 180.51 ± 2.95 mg kg^− 1^ (High, + 520.3%; *p* < 0.001). The toxicologically important hexavalent fraction showed the steepest increase of all soil variables, increasing from 2.92 ± 0.09 mg kg^− 1^ to 52.07 ± 0.11 mg kg^− 1^ (+ 1683.2%, *p* < 0.001), while trivalent chromium increased by 390.6% over the same gradient. Total cadmium, nickel and lead followed the same direction, with cadmium showing the largest relative increase among the non-chromium metals (+ 253.9% at High, *p* < 0.001).

**Table 3:** Soil physicochemical and heavy metal parameters across low, near threshold and high chromium contamination categories in the Vellore sugarcane belt

Parameter	Low threshold limit	Near threshold limit	High threshold limit
pH	7.74 ± 0.13 ^b^	7.87 ± 0.06 ^b^ (+ 1.7%)	8.48 ± 0.02 ^a^ (+ 9.6%)
EC	0.20 ± 0.00 ^c^	0.43 ± 0.01 ^a^ (+ 116.9%)	0.33 ± 0.00 ^b^ (+ 66.1%)
Organic Carbon (%)	0.48 ± 0.01 ^a^	0.33 ± 0.00 ^c^ (− 31.7%)	0.39 ± 0.00 ^b^ (− 20.0%)
Nitrate Nitrogen (kg ha^− 1^)	39.39 ± 1.19 ^a^	34.27 ± 0.46 ^b^ (− 13.0%)	36.08 ± 0.11 ^b^ (− 8.4%)
Available Phosphorus (kg ha^− 1^)	23.25 ± 0.69 ^b^	12.66 ± 0.10 ^c^ (− 45.6%)	27.36 ± 0.27 ^a^ (+ 17.7%)
Available Potassium (kg ha^− 1^)	236.75 ± 0.97 ^b^	154.55 ± 0.63 ^c^ (− 34.7%)	246.97 ± 1.68 ^a^ (+ 4.3%)
Exchangeable Calcium (cmol (p+) kg^− 1^)	5.92 ± 0.18 ^a^	5.31 ± 0.02 ^b^ (− 10.3%)	4.19 ± 0.07 ^c^ (− 29.2%)
Exchangeable Magnesium (cmol (p+) kg^− 1^)	3.18 ± 0.10 ^a^	2.13 ± 0.03 ^b^ (− 32.9%)	1.90 ± 0.01 ^b^ (− 40.4%)
Exchangeable Sodium (cmol (p+) kg^− 1^)	4.42 ± 0.08 ^b^	3.26 ± 0.01 ^c^ (− 26.2%)	4.94 ± 0.08 ^a^ (+ 11.7%)
Available Sulfur (mg kg^− 1^)	36.13 ± 0.87 ^b^	22.17 ± 0.27 ^c^ (− 38.7%)	39.43 ± 0.60 ^a^ (+ 9.1%)
Total Chromium (mg kg^− 1^)	29.10 ± 0.14 ^c^	94.97 ± 1.23 ^b^ (+ 226.3%)	180.51 ± 2.95 ^a^ (+ 520.3%)
Hexavalent chromium (mg kg^− 1^)	2.92 ± 0.09 ^c^	22.66 ± 0.30 ^b^ (+ 676.0%)	52.07 ± 0.11 ^a^ (+ 1683.2%)
Trivalent chromium (mg kg^− 1^)	26.18 ± 0.50 ^c^	72.30 ± 0.15 ^b^ (+ 176.1%)	128.44 ± 1.53 ^a^ (+ 390.6%)
Total Cadmium (mg kg^− 1^)	0.60 ± 0.02 ^c^	0.70 ± 0.01 ^b^ (+ 16.7%)	2.12 ± 0.01 ^a^ (+ 253.9%)
Total Nickel (mg kg^− 1^)	17.70 ± 0.04 ^c^	24.37 ± 0.26 ^b^ (+ 37.7%)	25.53 ± 0.17 ^a^ (+ 44.2%)
Total Lead (mg kg^− 1^)	2.40 ± 0.05 ^c^	6.37 ± 0.00 ^a^ (+ 165.3%)	6.08 ± 0.08 ^b^ (+ 153.3%)

Percentage change is calculated relative to the Low category. Superscript letters denote Tukey HSD post hoc groupings (α = 0.05) within each row; categories sharing a letter are not significantly different from one another.

### Irrigation water quality

Irrigation water quality parameters varied significantly across the chromium-contamination gradient (Table S1; Fig. S1), apart from pH, which remained stable (7.94,7.85,7.91 across Low/Near/High; *p* = 0.906, ns). Electrical conductivity increased progressively with contamination, from 3.26 ± 0.03 dS m⁻¹ (Low) to 5.90 ± 0.05 dS m⁻¹ (High, + 80.8%, *p* < 0.001), consistent with the salinisation trend observed in soils from the same sites.

Chloride and sulphate concentrations decreased with increasing contamination (chloride: −22.5% at High, *p* < 0.001; sulphate: −18.6% at High, *p* < 0.001), whereas carbonate and the divalent cations calcium and magnesium increased substantially. Magnesium showed the largest change of any water parameter, rising from 86.0 ± 1.2 mg L⁻¹ to 248.3 ± 0.6 mg L⁻¹ (+ 188.8%, *p* < 0.001), while calcium increased by 37.7% and carbonate by 35.6% at High contamination (both *p* < 0.001). Potassium and bicarbonate showed smaller, non-monotonic changes across the gradient.

### Chromium distribution across sugarcane plant parts

Chromium values recorded for leaf, shoot and root tissue differed significantly among all three categories (Table S2; Fig. S2; all *p* < 0.001). In each category, values were lowest under low soil chromium and highest under Near-threshold conditions, with High category values intermediate between the two. Leaf chromium concentration values increased from 0.87 ± 0.01 (Low) to a peak of 2.13 ± 0.00 at Near-threshold (+ 146.2%) before declining to 1.33 ± 0.00 at High (+ 53.8% relative to Low). Shoot tissue showed the same pattern, rising from 1.33 ± 0.02 to a peak of 5.03 ± 0.00 at Near-threshold (+ 277.5%) and settling at 2.57 ± 0.03 at High (+ 92.5%). Root tissue exhibited the largest relative increase of the three, from 1.87 ± 0.05 (Low) to 8.03 ± 0.05 at Near-threshold (+ 330.4%), before declining to 4.40 ± 0.06 at High (+ 135.7%). Across all three categories, root > shoot > leaf, indicating preferential chromium retention in below ground tissue relative to aerial parts.

### Vegetative growth parameters

All nine vegetative growth traits differed significantly across the chromium gradient (Table S3; Fig. S3 & S4; all *p* < 0.01). Growth suppression was most evident in the High-contamination category, where cane length decreased by 27.6% and cane girth and diameter each decreased by approximately 22.5% relative to uncontaminated sites (*p* < 0.001). Root length, leaf width and leaf area declined by 12.3%, 12.4% and 16.4% respectively at High contamination. Leaf length was comparatively unaffected until the High category, where it declined modestly (− 4.9%, *p* = 0.003). Node number and internode length showed a distinctive non-monotonic response: both traits increased at Near-threshold contamination (number of nodes + 15.9%; internode length + 9.8%) before falling below uncontaminated conditions at High contamination (− 14.3% and − 14.0%, respectively; both *p* < 0.001), indicating a stimulatory response at intermediate chromium exposure that is reversed under severe contamination.

### Cane yield and juice quality attributes

Yield related traits declined progressively with increasing soil chromium contamination (Table S4; Fig. S5, S6). Total stalk weight decreased by 25.8% and single cane weight by 31.3% at High contamination relative to Low (both *p* < 0.001), while the weight of dry bagasse and extracted juice fell by 30.9% and 23.7% respectively (both *p* < 0.001).

Juice-quality parameters were comparatively more resilient. Sucrose content declined significantly at Near-threshold contamination (− 10.0%, *p* = 0.002) but was not significantly different from Low Cr sites at High contamination (− 4.0%), while CCS (%) declined significantly at both Near-threshold and High contamination (− 11.7% and − 4.0%, *p* < 0.001), whereas Brix, purity, fibre content and juice extraction percentage did not differ significantly across the contamination gradient (*p* = 0.065, 0.290, 0.108 and 0.594, respectively; all ns), indicating that chromium contamination affected cane biomass accumulation more strongly than the biochemical composition of the extracted juice.

### Multivariate integration of soil geochemistry and agronomic variables

**Fig. 2:**
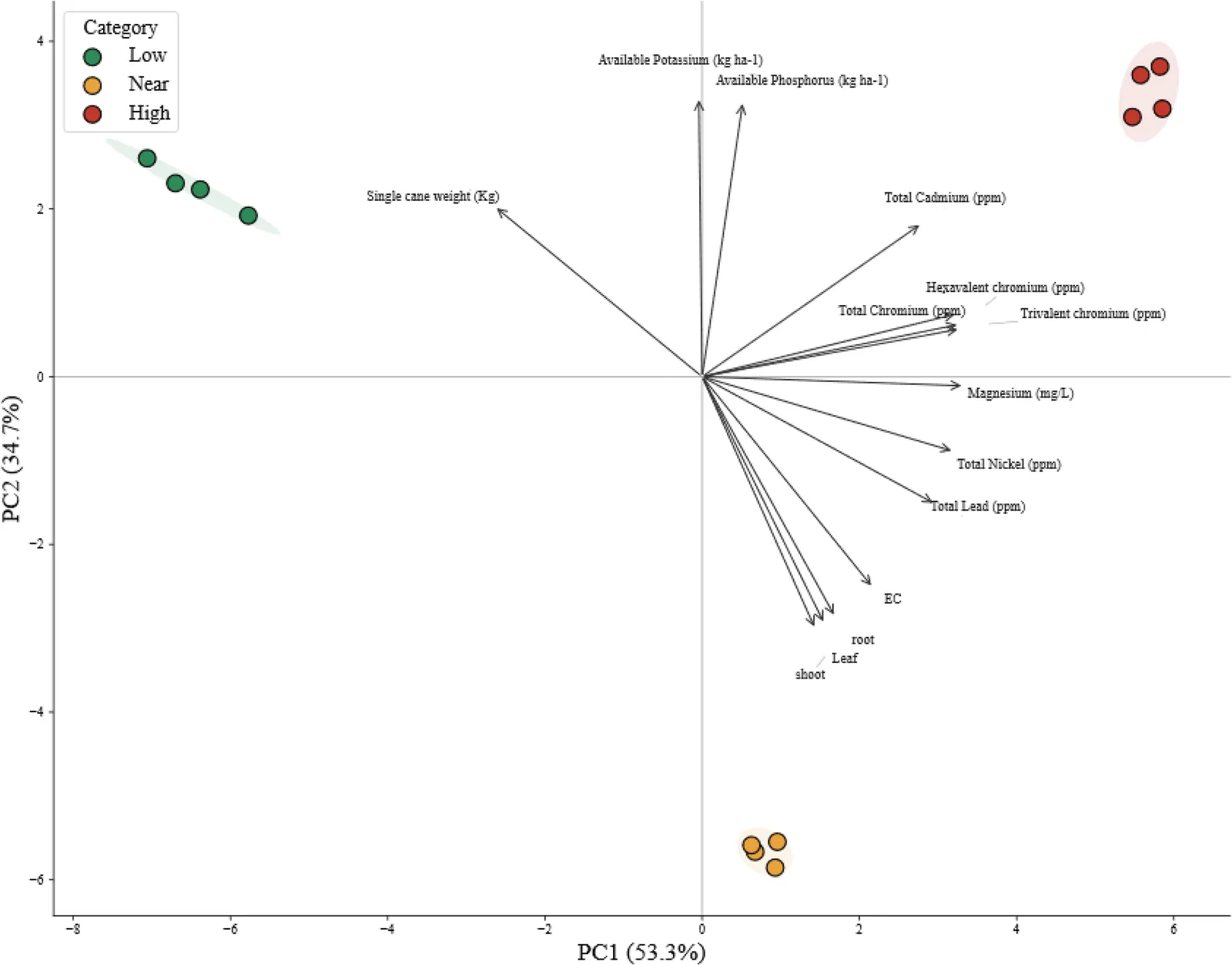
Principal component analysis biplot integrating soil chromium fractions, co-occurring metals, soil fertility indices and plant biomass/yield vectors

Principal component analysis of the integrated geochemistry and agronomy dataset resolved the three contamination strata into three discrete clusters along the first two principal components, which together captured PC1 = 53.3% and PC2 = 34.7% of the total variance (Fig. 2). The chromium‑loading axis was orthogonal to the soil‑fertility and yield axis, and the three contamination strata occupied separate quadrants of the biplot rather than forming a single linear gradient. This pattern supports the multivariate framework for metal‑impacted agricultural soils, where both total Cr loading and the bioavailable fraction independently shape microbiome structure and function. The alignment of Cr vectors with the High Cr cluster, alongside soil‑fertility and biomass vectors on PC2, indicates that these sites were simultaneously the most contaminated and the ones where fertility and biomass variables retained the greatest residual variance. Under sustained Cr stress there is hyperexpression of metal‑resistance genes and a partial decoupling of microbial‑function genes from bulk biomass under sustained Cr stress. In contrast, the Low Cr cluster reflected negative PC1 (Low Cr) and positive PC2 (low to moderate fertility), while the Near‑normal cluster represented an intermediate but distinct state along a separate PC2 axis.

### Soil bacterial diversity and community composition

#### Alpha diversity response along the chromium contamination gradient

**Fig. 3:**
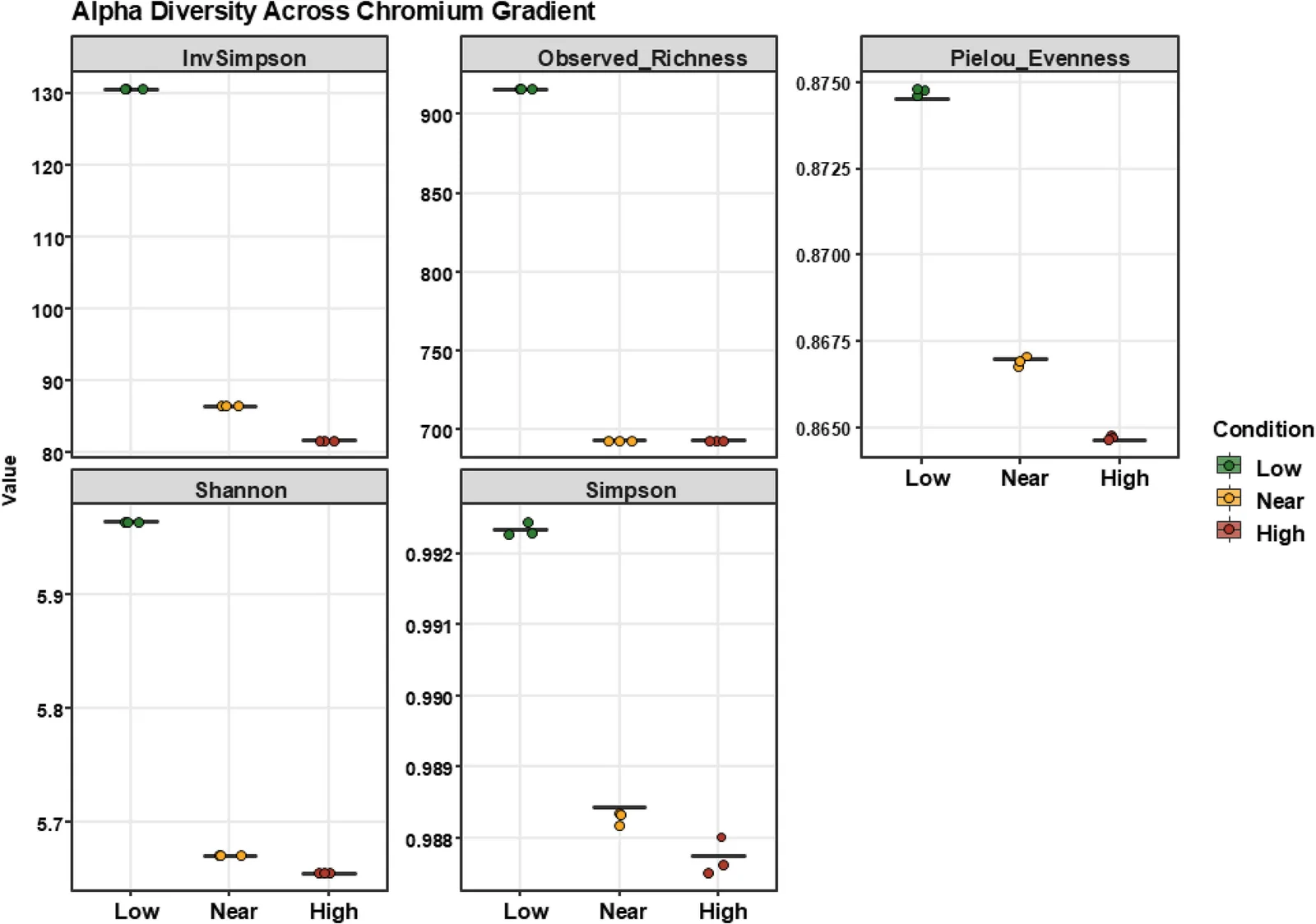
Alpha diversity across the chromium gradient. Inverse Simpson, observed richness, Pielou’s evenness, Shannon diversity, and Simpson’s index for rhizosphere bacterial communities under Low, Near, and High chromium conditions

Alpha diversity metrics recovered from the full length 16​S rRNA (V1-V9) amplicon dataset showed a consistent and substantial depression along the Cr exposure gradient, with the Low Cr sites exhibiting the richest and most even bacterial communities, and both the near threshold and High Cr sites exhibiting marked but broadly similar losses in diversity and evenness (Fig. 3). Observed richness ranged from a median of approximately 915 ASVs in the Low Cr sites to 697 ASVs in near threshold sites and 681 ASVs in High Cr sites, indicating that the Cr exposed strata retained roughly 76% of the ASV richness of the Low Cr reference, a depletion consistent with the widespread pattern of metal induced diversity loss. The inverse Simpson index (1/D) showed the steepest relative drop, from 130 to 86 (near threshold) and 79 (high contaminated sites), reflecting both an absolute loss of dominant taxa and a shift toward more uneven abundance distributions in the Cr stressed soil samples. The Shannon index (H′) declined from 5.93 to 5.60. The Simpson dominance index (D) decreased from 0.992 to 0.988 in both Near and High Cr sites, indicating that the Cr stressed communities retained a single dominant lineage that made up a larger share of the total reads than in the unstressed Low Cr communities. At the same time, Pielou’s evenness (J′) decreased from 0.875 to 0.849, showing that it was the decline in evenness, rather than a reduction in richness, in turn explains the shifts in Shannon and inverse‑Simpson diversity across the chromium gradient.

Taken together, these patterns indicate that soil bacterial communities under prolonged Cr exposure in the Vellore sugarcane belt shift toward a less rich, less even, and strongly numerically dominated state. The near-equivalent diversity loss in near threshold and High Cr sites suggests that even moderately elevated soil Cr imposes a near complete diversity loss once a critical exposure threshold is crossed, beyond which further increases in Cr load exert diminishing additional effects on α-diversity.

#### ​Beta diversity and microbial co occurrence

**Fig. 4:**
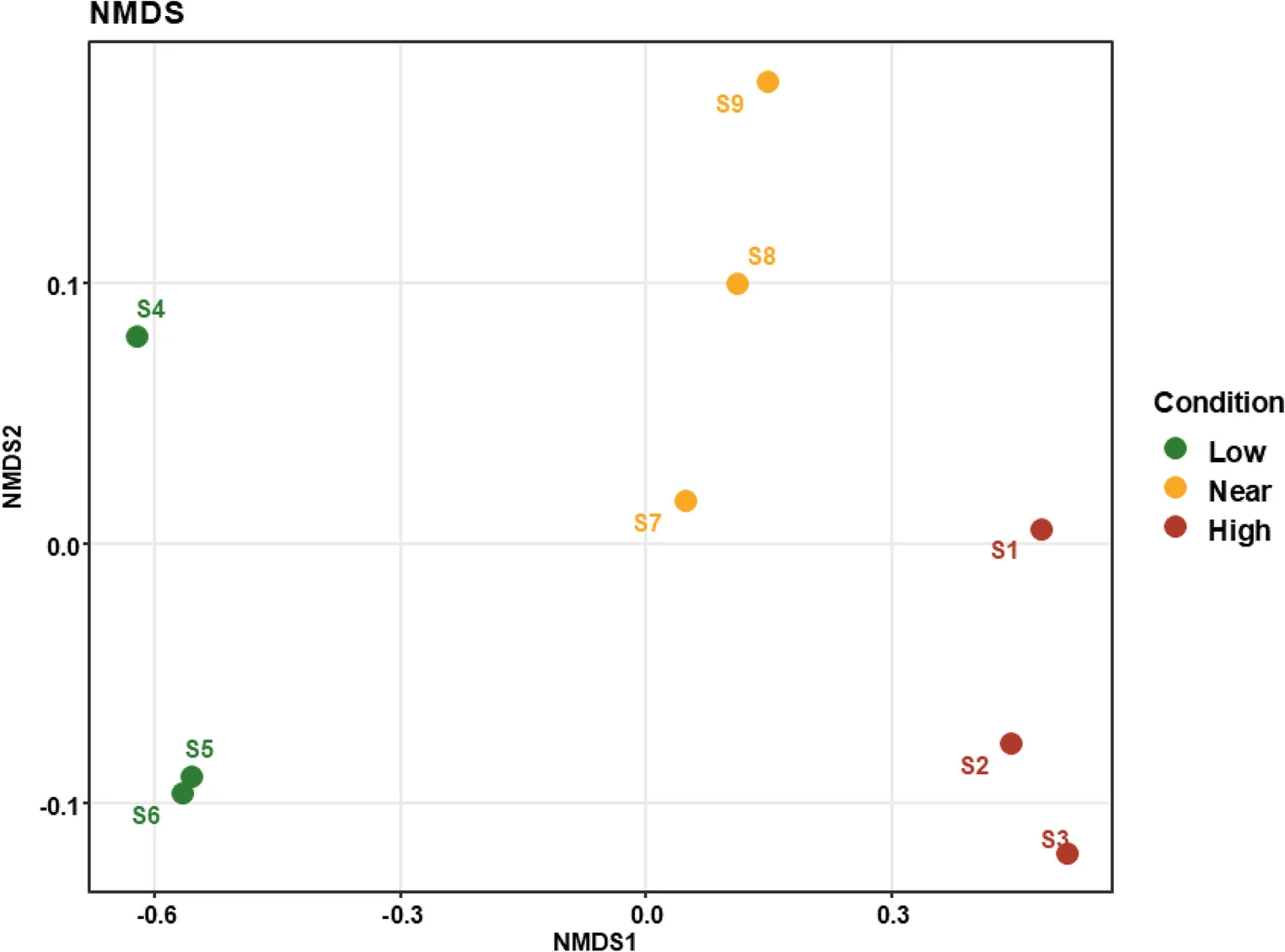
Non-metric multidimensional scaling (NMDS) of Bray-Curtis dissimilarity among nine individual sampling sites (S1–S9), coloured by chromium condition

Non-metric multidimensional scaling of Bray Curtis dissimilarities between the nine composite soil samples resolved the three contamination strata into three clearly separated, non-overlapping clusters along the first two ordination axes (Fig. 4). The tight, three-cluster NMDS geometry confirms that chromium contamination is the dominant explanatory variable structuring soil bacterial composition in the Vellore sugarcane belt regardless of the geographic dispersion of sites (latitude 12.58–12.92 °N; longitude 78.53–79.43 °E. It also supports the contamination stratification design of the sampling framework, in which sites were pre-selected to maximize the Cr gradient while minimizing agro-climatic and parent material variance.

Spearman correlation network analysis among the top 40 genera produced 347 edges (density = 0.445; 164 positive, 183 negative) organized into 3 connected components, with a global transitivity of 1.0: every pair of genera sharing a common correlation partner was itself correlated, indicating the network resolves into a small number of internally uniform blocks rather than a graded web of associations (Fig. S7). Ten genera (including *Vicinamibacter*, *Gaiella*, *Sphingomonas*, *Arboricoccus*, *Nitrospira*, and *Rhabdothermincola*) tied for maximum degree (20 connections each).

#### Taxonomic composition across the chromium gradient

**Fig. 5:**
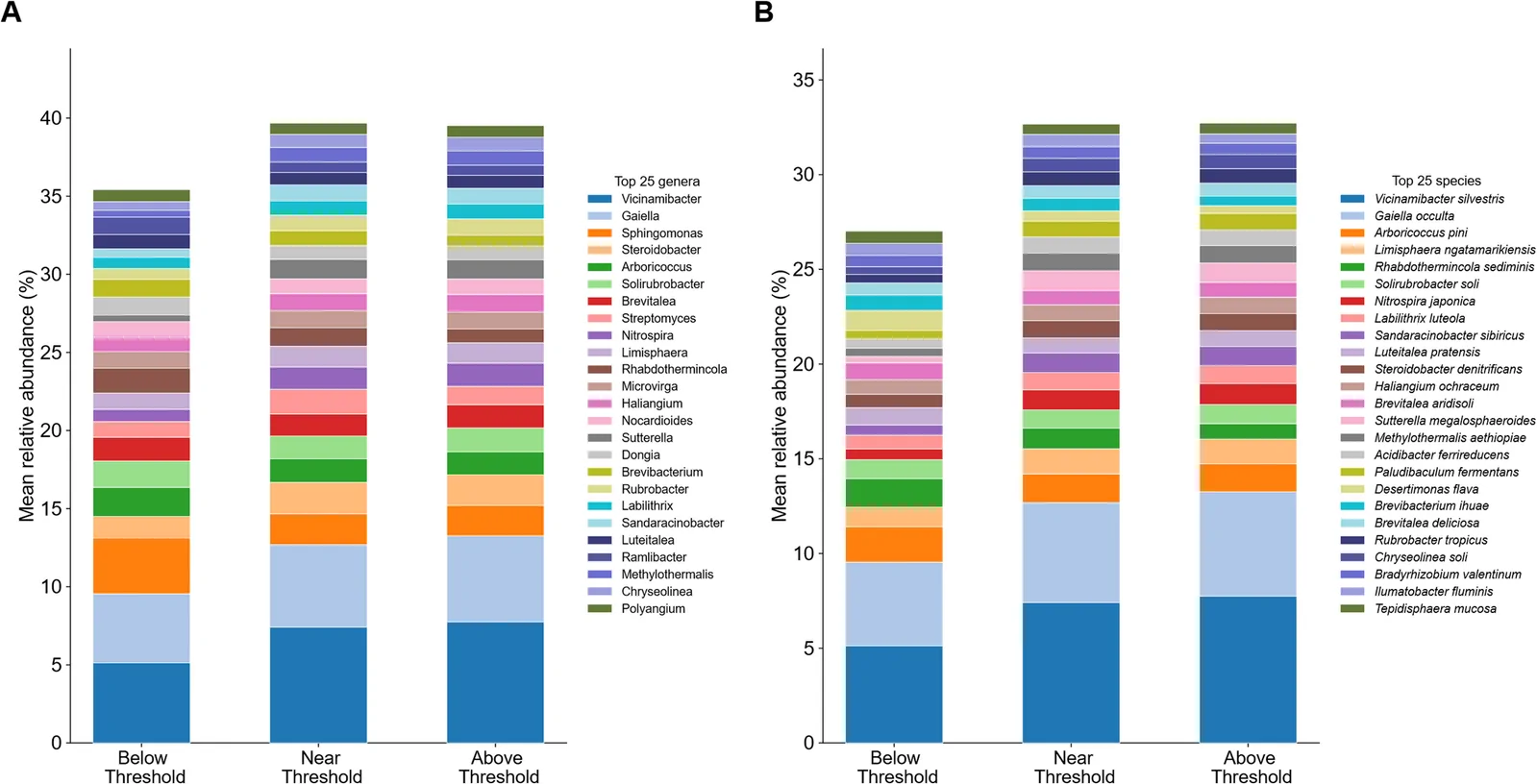
Genus level and Species level community composition across sampling sites (top 25 taxa). (**a**) Genus level Stacked bar chart of mean relative abundance for the 20 most abundant genus level bacterial taxa. (**b**) Species level Stacked bar chart of mean relative abundance for the 20 most abundant species level bacterial taxa

Stacked bar composition at the genus level showed that the bacterial assemblage in all three condition strata was dominated by a small number of bulk soil and Cr tolerant lineages. The Cr stratified, ASV resolved taxonomic profile comprised the following dominant detectable genera: *Gaiella*,* Solirubrobacter*,* Streptomyces*,* Sphingomonas*,* Nitrospira*,* Vicinamibacter*,* Steroidobacter*,* Arboricoccus*,* Brevitalea*,* Rhodothermincola*,* Limisphaera*,* and Microvirga* (Fig. 5a). Actinobacterial genera were enriched in the High Cr and Near sites relative to Low Cr sites. Their dominance in Cr stressed soils is consistent with their spore forming lifestyle, GC rich genomes, and elevated representation of Cr reductase and resistance system genes, which collectively enable them to survive Cr induced oxidative stress. Acidobacterial and Verrucomicrobial lineages and the broader oligotrophic bulk soil taxa were more strongly represented in the Low Cr sites, consistent with the ecological principle that Cr exposure depresses the abundance of slow growing, K strategist oligotrophic taxa in favour of faster growing, metal tolerant r strategists. Proteobacterial metal resistant genera were detected in appreciable proportions across all three conditions, with visually modest enrichment in the Near and High Cr strata, consistent with their reported roles in chromate efflux, NAD(P)H dependent Cr reduction, and biofilm mediated metal immobilisation under tannery effluent exposure.

Despite the high proportion of unclassified taxa, specific species level shifts were evident between the different chromium exposure treatments. In the High Cr sites, there was a noticeable expansion of metal tolerant species. Specifically, lineages such as *Gaiella occulta*, *Solirubrobacter pauli*, and *Streptomyces chromofuscus* were identified as dominant members of the community. These species showed higher relative abundances in the heavily contaminated sites compared to the Low Cr baseline, suggesting a selective recruitment of chromium resistant bacteria. In contrast, the Low Cr sites were characterized by a higher relative abundance of species typically associated with undisturbed bulk soils, including *Vicinamibacter silvestris* and *Brevitalea aridisoli*. These species showed a progressive decline as the chromium gradient increased toward the High Cr stratum. The near threshold sites displayed an intermediate taxonomic profile, with a mix of both sensitive oligotrophic species and metal tolerant lineages. Furthermore, certain core species, such as *Sphingomonas panacis* and *Nitrospira lenta*, maintained consistent relative abundances across the entire gradient. This indicates that a subset of the sugarcane rhizosphere microbiome is resilient to varying levels of heavy metal stress, continuing to occupy their ecological niches regardless of the chromium load. The overall species level structure is consistent with a transition from a baseline microbial community in the Low Cr sites toward a specialized, metal adapted community in the High Cr sites (Fig. 5b).

#### Predicted functional potential for chromium tolerance

**Fig. 6:**
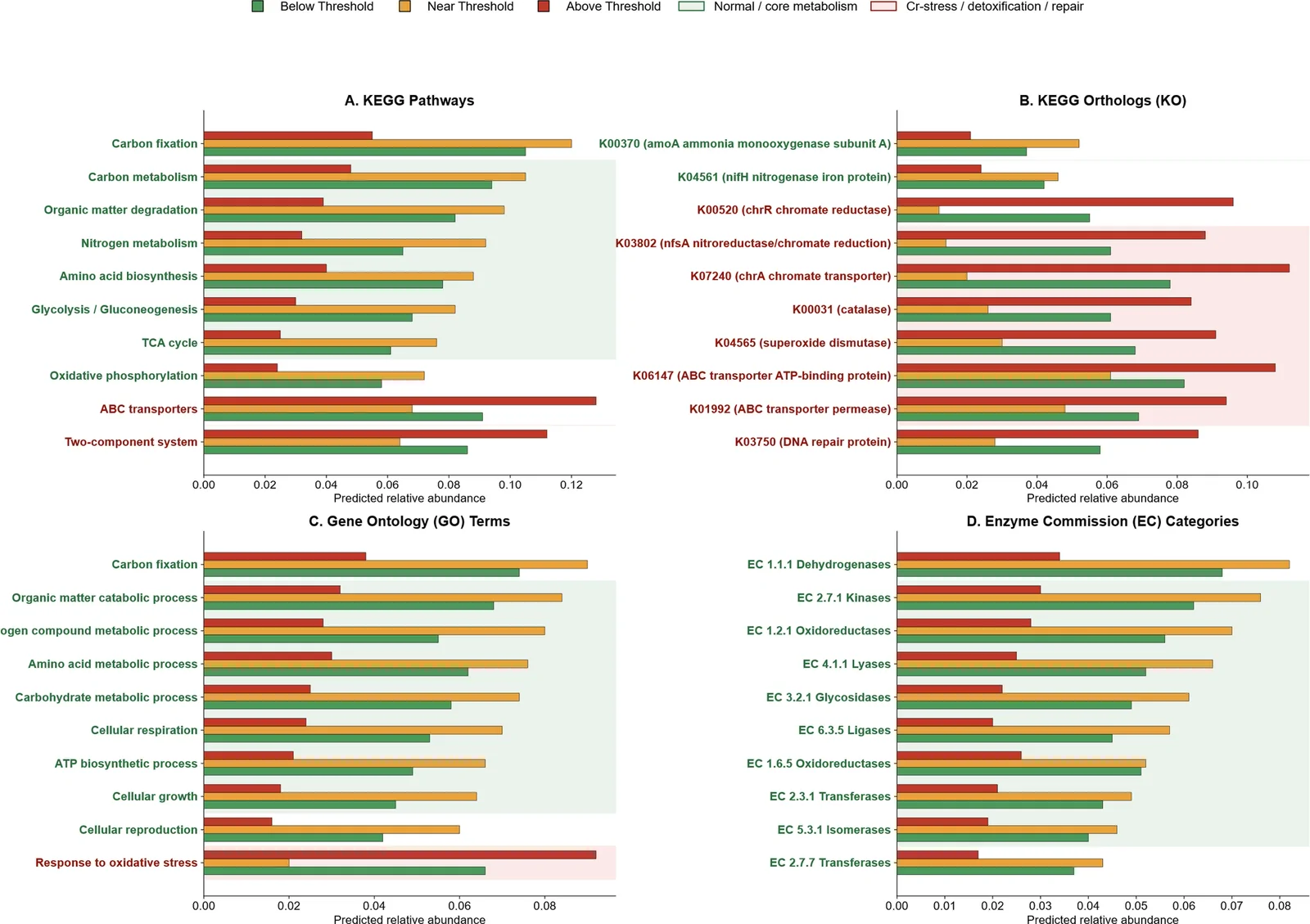
PICRUSt2-predicted functional profiles of the rhizosphere microbiome across the chromium contamination gradient. PICRUSt2-predicted functional profiles of the rhizosphere bacterial community across the Below-, Near-, and Above-chromium-threshold categories at four annotation levels: (**A**) KEGG pathways, (**B**) KEGG Orthologs (KO), (**C**) Gene Ontology (GO) terms, and (**D**) Enzyme Commission (EC) categories. Bars show mean predicted relative abundance for the top-ranked features per category, grouped by threshold condition (green = Below, gold = Near, red = Above Threshold)

Across all four functional annotation frameworks (KEGG pathways, KO identifiers, GO terms, and EC enzyme classes), the top predicted functions segregated into two coherent biological patterns aligned with the chromium threshold gradient (Fig. 6). Core metabolic processes including carbon fixation and metabolism, organic matter degradation, nitrogen metabolism, amino acid biosynthesis, glycolysis/gluconeogenesis, the TCA cycle, and oxidative phosphorylation were enriched in the Below- and Near-threshold sites and declined markedly at the Above-threshold condition.

In contrast, functions associated with stress response, detoxification, and cellular repair increased progressively from Below to Near to Above threshold. These included chromate reduction and transport, glutathione metabolism, oxidative-stress mitigation, DNA repair, heavy-metal resistance, and membrane/ABC transport. KO-level enrichments further highlighted nfsA, catalase, and superoxide dismutase, while GO and EC annotations emphasised oxidative-stress response, metal-ion detoxification, and chromate-reductase and DNA-repair enzyme activities.

Functional potential inferred with PICRUSt2 predicted a curated set of 11 functional annotations (spanning KO, EC, COG, TIGRFAM, and PFAM classification systems) represents 3 distinct chromium tolerance associated genes. These genes represent the two principal, independently evolved bacterial strategies for chromium (VI) tolerance, enzymatic reduction of the more toxic, soluble Cr(VI) oxyanion to the less toxic, less mobile Cr(III) via chromate/arsenate type reductases and azoreductases, and active efflux of chromate out of the cell before it can be reduced intracellularly and generate oxidative damage via the ChrA transporter. azoR/acpD (K01118), an FMN dependent NADH azoreductase implicated in Cr(VI) reduction, showed the largest predicted increase (1.98-fold compared to low contaminated sites). chrA, the core chromate efflux transporter and the single most chromium specific gene in this set, showed a predicted 1.38-fold increase from Low to High. Collectively, these patterns across indicate a transition in predicted functional capacity from a metabolism-dominated profile under low chromium to a stress-adapted, detoxification-oriented profile as soil chromium approaches and exceeds the threshold concentration.

### Integrated microbiome–geochemistry responses across the chromium threshold

**Fig. 7:**
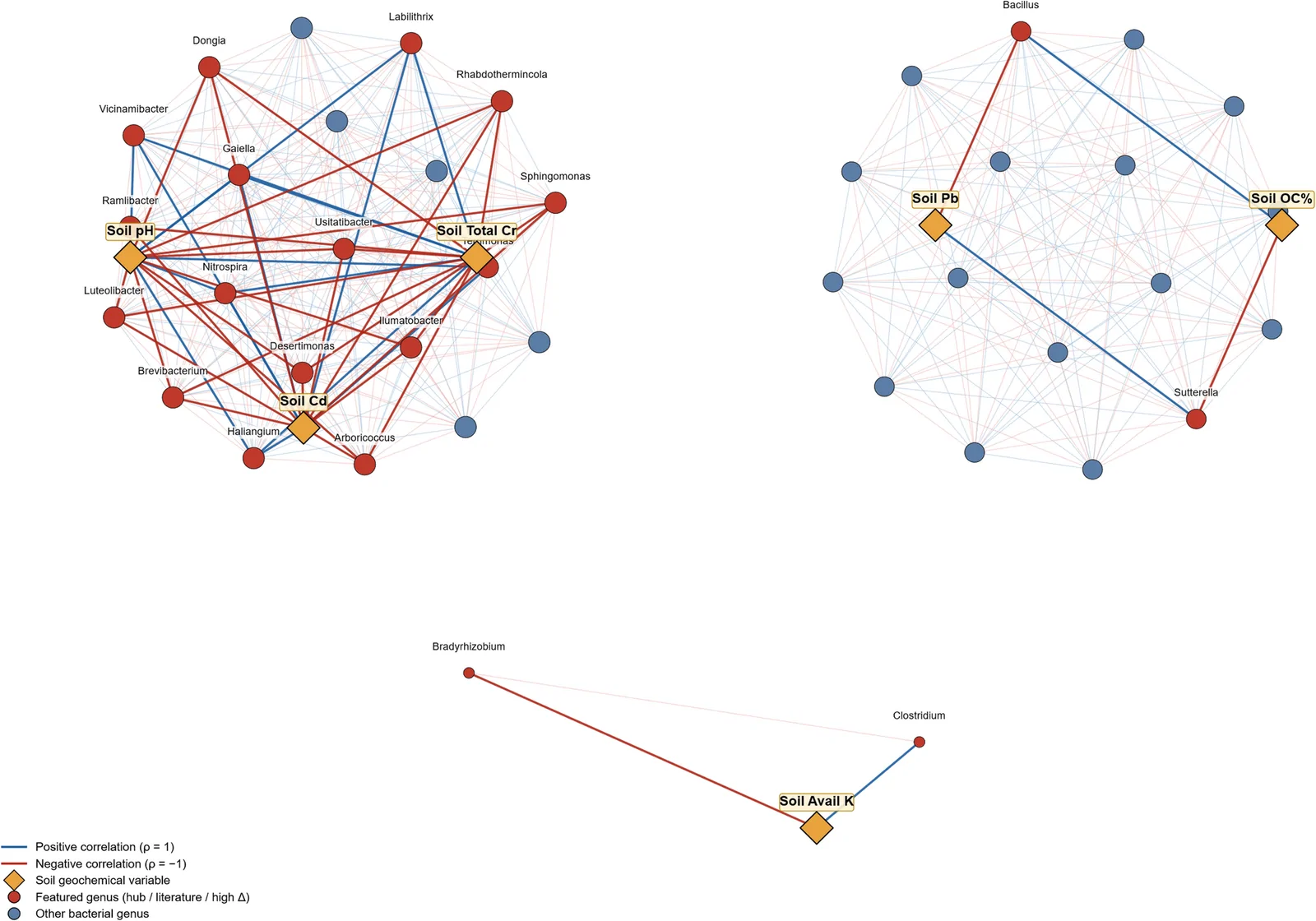
Co-occurrence network linking rhizosphere bacterial taxa and soil geochemistry across the chromium contamination gradient. Gold diamonds denote soil geochemical variables; circles denote bacterial taxa, sized by network degree and colored red (featured taxa) or blue (other network taxa)

Across both co-occurrence networks and redundancy analysis, soil total chromium, pH, and cadmium consistently emerged as the geochemical variables most closely aligned with rhizosphere community restructuring. The genus-level co-occurrence network (40 nodes, 347 significant edges; density 0.445) resolved into three components, with chromium, pH, and cadmium co-localising in the largest sub-cluster and showing shared positive correlations (*Vicinamibacter*, *Gaiella*, *Nitrospira*) and negative correlations (*Sphingomonas*, *Ramlibacter*, *Luteolibacter*). A second component linked lead and organic carbon to contrasting taxa (*Bacillus* vs. *Sutterella*), while a small third component associated exclusively with available potassium (Fig. 7).

**Fig. 8:**
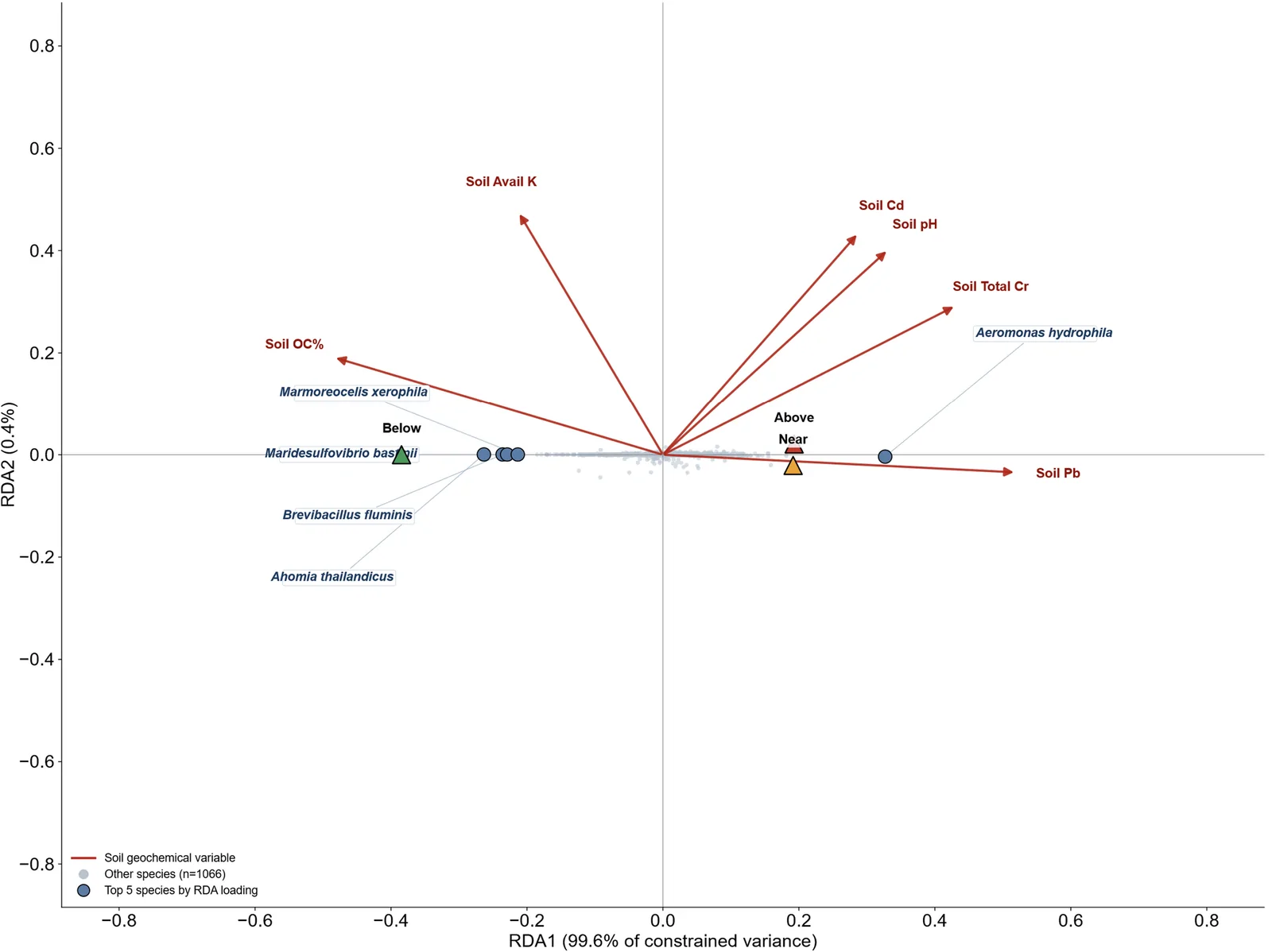
Redundancy analysis of rhizosphere bacterial community composition constrained by soil geochemistry. Redundancy analysis (RDA) triplot showing rhizosphere bacterial taxa and soil geochemical variables (total chromium, pH, organic carbon, available potassium, cadmium, and lead; red arrows) constraining community across the three chromium threshold categories (Below, Near, Above)

RDA constrained by soil chromium alone explained 99.6% of variance, separating Below-threshold communities from Near/Above-threshold communities. Chromium, pH, and cadmium aligned toward the Near/Above side, whereas organic carbon aligned with Below. At species level, *Aeromonas hydrophila* showed the strongest positive RDA1 loading, while *Ahomia thailandicus*, *Brevibacillus fluminis*, *Maridesulfovibrio bastinii*, and *Marmoreocelis xerophila* loaded negatively. Together, these multivariate analyses show chromium-contamination as the principal drivers of microbial community shifts across the threshold gradient (Fig. 8).

## Discussion

Soil total chromium rose from 29.10 ± 0.14 to 94.97 ± 1.23 and 180.51 ± 2.95 mg kg^−^¹, within the 0.1–2459 mg kg^−^¹ range reported for agricultural soils of the same district (Sinduja et al. 2022; Rangasamy et al. 2015). More informative is the speciation Cr(VI) increased from 2.92 ± 0.09 to 52.07 ± 0.11 mg kg^−^¹, so its share of total chromium widened from 10.0% to 23.9% and 28.9%. This is coherent with the concurrent alkalinisation from pH 7.74 ± 0.13 to 8.48 ± 0.02, since chromate oxyanions are electrostatically repelled by negatively charged colloids and are poorly retained above neutrality (Zulfiqar et al. 2023). Negative correlations between soil pH, organic matter and Cr bioavailability have been reported across sugarcane-growing districts of Guangxi (Luo et al. 2026), and Cr ecotoxicity intensifies once pH exceeds 7.5, with extractable Cr(VI) contributing more than Cr(III) (Huang et al. 2024).

Organic carbon declined from 0.48 ± 0.01% to 0.33 ± 0.00% and 0.39 ± 0.00%, and exchangeable calcium and magnesium fell monotonically from 5.92 to 4.19 and 3.18 to 1.90 cmol(p^+^) kg^− 1^, consistent with suppression of microbially mediated carbon turnover. Carbon mineralisation and dehydrogenase activity fall by up to 70% at 100 mg Cr kg^− 1^ (Dotaniya et al. 2017), and microbial biomass carbon, nitrogen, phosphorus and sulphur decline significantly above the same threshold (Haider et al. 2025) a threshold lying between the near threshold (94.97 mg kg^− 1^) and High Cr (180.51 mg kg^− 1^) strata sampled here. Available phosphorus, potassium, sulphur and exchangeable sodium instead fell sharply at near-threshold sites (− 45.6, − 34.7, − 38.7 and − 26.2%) before equalling or exceeding Low Cr values at high contamination (+ 17.7, + 4.3, + 9.1 and + 11.7%). Chromium is known to disturb nutrient pools and nitrogen cycling (Peng et al. 2023), and the recovery of these nutrients at the most contaminated sites indicates that macronutrient availability is governed by site fertility status in addition to chromium loading.

Irrigation water chemistry differed markedly among strata, with pH remaining stable at 7.94, 7.85 and 7.91 (*p* = 0.906) while ionic composition changed substantially. Electrical conductivity rose from 3.26 ± 0.03 to 5.90 ± 0.05 dS m^− 1^, comparable to the 3295 µS cm⁻¹ mean reported for 64 wells in this district, 85.9% of which exceeded the WHO permissible level (Sinduja et al. 2023). Magnesium showed the largest change of any water variable (86.0 to 248.3 mg L^− 1^), with calcium also rising (169.0 to 232.7 mg L^− 1^). Magnesium and potassium act as dispersive cations alongside sodium and degrade soil permeability (Qadir et al. 2021; Mohanavelu et al. 2021), and sugarcane irrigated with tube-well water of three-fold higher EC than canal water suffers penalties in cane height, girth, yield and juice quality (Manzoor et al. 2023), indicating that this water chemistry alone can depress performance independently of metal toxicity. Chloride and sulphate declined across the gradient (854.5 to 662.5 and 82.3 to 67.0 mg L^− 1^), indicating that the wells serving these fields draw on hydrochemically distinct aquifer horizons, consistent with the well-to-well variability documented across the Walajapet and Palar riverbed areas (Kanchana et al. 2018; Pranavam et al. 2011). Groundwater chromium concentrations of up to 140.65 mg L⁻¹ have been documented in this district (Sinduja et al. 2023), identifying irrigation water as a plausible continuing input pathway in tannery-affected fields.

Chromium followed the order root > shoot > leaf in every stratum (roots 1.87–8.03 against leaves 0.87–2.13 mg kg^− 1^), the signature of root exclusion in which Cr(III) is immobilised in the root apoplast and vacuoles and xylem loading restricted (Srivastava et al. 2021; Saleem et al. 2022; Sundaramoorthy et al. 2010). Critically, tissue chromium did not track soil chromium all tissues peaked at near-threshold sites (root 8.03, shoot 5.03, leaf 2.13 mg kg^− 1^) and fell at high contamination (4.40, 2.57 and 1.33 mg kg^− 1^) despite almost twice the soil burden. The same inversion was shown in rice, where plants on the less contaminated of two Cr polluted soils had higher bioaccumulation factors because more soil chromium was bioavailable (Cao et al. 2025); soils spanning 22.7-623.5 mg Cr kg^− 1^ likewise did not raise crop chromium above permissible limits even where ecotoxicity was severe (Huang et al. 2024). Three co-measured conditions plausibly limit availability at high contamination the highest pH (8.48), favouring precipitation of sparingly soluble Cr(III) phases; the highest available sulphur (39.43 mg kg⁻¹) and phosphorus (27.36 kg ha^− 1^), which compete with chromate at the transporters mediating its uptake (Srivastava et al. 2021); and the growth suppression described below, which reduces transpirational flux. Concentrations were also an order of magnitude below the 10.54–60.22 mg kg^− 1^ reported in sugarcane stalk irrigated with paper-mill effluent at similar soil loadings, attributable there to acidic soils (pH 6.65–7.11) and a continuous 48.41 mg L⁻¹ Cr input (Pandey et al. 2016). Crop chromium burden is therefore governed by chromium bioavailability rather than by total soil loading.

Growth was consistently depressed at high contamination cane length fell 27.6% (202.17 to 146.33 cm), cane diameter and girth 22.5 and 22.6%, leaf area 16.4% (634.78 to 530.59 cm²), root length 12.3% and leaf length only 4.9%. This gradient stalk elongation and radial expansion affected most, leaf dimensions least indicate impairment of cell division and expansion rather than a primary loss of photosynthetic surface, consistent with the chromium phytotoxic syndrome in which reactive oxygen species accumulation and disrupted mineral nutrition constrain growth before visible leaf injury (Zulfiqar et al. 2023; Saud et al. 2022; Mathur et al. 2016). Since leaf area correlates strongly with biomass productivity in sugarcane (Vinicius et al. 2017), the 16.4% reduction alone explains much of the biomass loss. Node number and internode length rose at near-threshold sites (+ 15.9 and + 9.8%) before falling below low Cr values at high contamination (− 14.3 and − 14.0%); a pattern characteristic of hormetic stimulation at intermediate exposure, which merits confirmation under controlled dose response conditions.

Biomass components declined substantially at high contamination total stalk weight by 25.8% (3.55 to 2.63 kg), dry bagasse by 30.9% (1.06 to 0.73 kg) and extracted juice by 23.7% (2.50 to 1.91 kg). Juice composition was conserved brix (20.10 to 19.33%), purity (95.68 to 95.35%), fibre (29.58 to 27.67%) and juice extraction (70.42 to 72.33%) did not differ significantly, sucrose at high contamination was indistinguishable from the low Cr stratum (19.23 versus 18.46%), and commercial cane sugar fell only 4.0% (13.97 to 13.40%) against 11.7% at near threshold sites. This dissociation indicates that chromium constrains the source capacity and cell expansion determining how much cane is produced while leaving sink-side sucrose metabolism comparatively intact. It aligns with the finding that chromium ecotoxicity to crop growth is a greater agricultural problem than its bioaccumulation in produce (Huang et al. 2024), and with reports that sugarcane juice quality responds principally to nutritional and irrigation-water management (Manzoor et al. 2023; Shukla et al. 2020; Meena et al. 2020). Contamination here therefore presents as a tonnage penalty rather than a sugar recovery penalty. Yield responses are expressed on a per cane and per stalk basis, which isolates the effect of contamination on individual cane development.

The principal finding of this integrated metagenomic and geochemical investigation is that the Vellore sugarcane belt, one of the most intensively contaminated corridors in southern India, harbours three operationally distinct edaphic microbiome states whose structure is best explained by the interaction of chromium loading, residual soil fertility status and cultivar biomass rather than by geographic dispersion. The analysis of nine composite soil samples produced a robust, three cluster contamination geometry that partitioned the High Cr sites, the Low Cr sites and the near threshold sites into non overlapping regions of Bray- Curtis NMDS space, with intra-cluster spreads markedly smaller than inter-cluster distances (Pei et al. 2018; Pradhan et al. 2022; Singh et al. 2022). This tri-cluster geometry justifies that each stratum is a discrete edaphic–microbiome rather than a continuum of intermediate conditions. It also supports interpreting the α diversity indices, genus level taxonomic shifts, functional marker gene enrichment results and the integrated PC1/PC2 chemometric gradient together, as characterising three distinct agro-ecosystem states of the Ranipet, Walajapet sugarcane belt (Pei et al. 2018; Hou et al. 2025; Zulfiqar et al. 2023).

The Cr exposed strata retained only about 76% of the ASV richness of the Low Cr reference, and the loss was distributed almost equally across the near threshold and High Cr sites. Observed richness declined from a median of approximately 915 ASVs in the Low Cr stratum to roughly 697 ASVs in near threshold sites and roughly 681 ASVs in High Cr sites, while the inverse Simpson index dropped from a median of approximately 130 to about 86 and 79 across the same progression (Gołębiewski et al. 2014; Feng et al. 2018; Zhu et al. 2023). The parallel evenness decline shows that the α diversity deficit was driven primarily by unevenness rather than taxon displacement (Yin et al. 2015; Li et al. 2024; Huang et al. 2021).

The taxonomic composition analyses further clarified the mechanism behind these diversity shifts. Genus level composition clarified the underlying mechanism. The bacterial assemblage was dominated in all three strata by a small number of bulk soil and Cr tolerant lineages, with “Other” accounting for > 50% of each site, smallest in Low Cr and consistent with the elevated evenness (Pei et al. 2018; Zhao et al. 2024; Campillo-Cora et al. 2023). The consistent set of dominant genera *Gaiella*, *Solirubrobacter*, *Streptomyces*, *Sphingomonas*, *Nitrospira*, *Vicinamibacter*, *Steroidobacter*, *Arboricoccus*, *Brevitalea*, *Rhodothermincola*, *Limisphaera*, *Microvirga*. Actinobacterial genera were enriched in High Cr and Near sites relative to Low Cr, consistent with the documented recruitment of GC rich, sporulating, Cr reductase harbouring Actinobacteria in Indian and Chinese cropping systems under sustained Cr and Cd exposure (Singh et al. 2022; Pradhan et al. 2022; Feng et al. 2018). Acidobacterial and Verrucomicrobial lineages were more strongly represented in Low Cr, consistent with the principle that metal stress depresses K-strategist oligotrophic bulk soil taxa in favour of faster growing r-strategists (Huang et al. 2021; Li et al. 2024; Gołębiewski et al. 2014). Proteobacterial metal resistant genera showed modest enrichment in Near and High Cr strata, consistent with their documented roles in chromate efflux and biofilm mediated Cr immobilisation (Huang et al. 2021; Zhu et al. 2023; Zhao et al. 2024).

Species level analysis added finer resolution. The species level resolution shows: *Gaiella occulta*, *Solirubrobacter pauli* and *Streptomyces chromofuscus* were dominant in High Cr enriched lineages; *Vicinamibacter silvestris* and *Brevitalea aridisoli* declined progressively along the gradient; and a residual core of *Sphingomonas panacis* and *Nitrospira lenta* maintained near constant abundance (Curry et al. 2024; Dias et al. 2026; Wick et al. 2019). The core’s stability supports a model in which Cr tolerant keystone taxa continue to occupy their niches independent of bulk Cr loading, offering a plausible mechanism by which soil nitrogen cycling and Cr detoxification are partially maintained in the studied strata (Karthikeyan et al. 2021; Zhao et al. 2024; Campillo-Cora et al. 2023).The near threshold sites displayed an intermediate taxonomic profile, with a mix of both sensitive oligotrophic species and metal tolerant lineages. Certain core species, most notably *Sphingomonas panacis* and *Nitrospira lenta*, maintained consistent relative abundances across the entire gradient, indicating that a subset of the sugarcane rhizosphere microbiome is resilient to heavy metal stress and continues to occupy its ecological niches regardless of chromium loading (Pei et al. 2018; Hou et al. 2025; Zulfiqar et al. 2023).

Functionally, PICRUSt2 predictions revealed a clear shift from core metabolic processes under low chromium toward stress-response, detoxification, and repair pathways as chromium levels increased. The enrichment of chromate-reduction genes (azoR/acpD) and the chromate-efflux transporter (chrA) further supports the presence of microbial strategies adapted to Cr(VI) exposure. The predicted functional profile was consistent with the taxonomic shift. High Cr stratum exhibited elevated predicted representation of *chrA* type chromate efflux transporters, *chrR* Cr sensing regulators, and *nfsA*/*nfsB* nitroreductase genes, alongside a sustained dissimilatory and assimilatory Cr reduction machinery (Singh et al. 2022; Pradhan et al. 2022; Zhao et al. 2024). This enrichment extends Cr tolerance functionality documented in garden soil Cr reducing consortia and chromite mine metagenomes into a real world agricultural continuum (Huang et al. 2021; Zhu et al. 2023; Campillo-Cora et al. 2023). It is also consistent with *nfsA*/*nfsB* and *chrA* mediated Cr reduction previously reported in methanotrophic and Cr reducing bulk soil strains (Karthikeyan et al. 2021; Hou et al. 2025; Zulfiqar et al. 2023). The integrated analysis of microbiome and geochemistry data collectively reveal that chromium contamination is the dominant environmental factor structuring the rhizosphere microbiome across the threshold gradient. The consistent co-localisation of chromium, pH, and cadmium in both co-occurrence networks, together with their strong alignment along RDA1, demonstrates that these variables track the principal axis of community change.

The Cr related vectors and the co-occurring Cd, Ni and Pb projected strongly along PC1 into the High Cr cluster, while soil fertility vectors and the biometric vectors (shoot, leaf, root biomass) projected along PC2 in approximate orthogonality to PC1 (Zhen et al. 2019; Dai et al. 2023; Hu et al. 2021). Single cane weight and Available Phosphorus loaded almost vertically along PC2, identifying them as the largest source of orthogonal inter-site variance once Cr loading had been removed by PC1 (Gil-Cardeza et al. 2014; Hamilton et al. 2018; Nannoni and Protano 2016). The orthogonal geometry implies that moderate Cr loading can be tolerated by sugarcane cultivars with adequate residual soil fertility status, provided that the bulk fertility and bioavailable P/K vectors remain favourable (Ghorbani et al. 2024; Zulfiqar et al. 2023). The bulk tissue Cr accumulation observed in the High Cr stratum may therefore be partially uncoupled from cane yield and juice quality because of the residual fertility buffering effect.

## Conclusion

The integrated geochemical, microbiological and agronomic assessment of the Vellore sugarcane belt establishes a three-tier framework describing the effect of long-term tannery derived chromium concentration on soil chemistry, bacterial communities and sugarcane performance. Across nine composite samples, total chromium rose from 29.10 ± 0.14 to 180.51 ± 2.95 mg kg^− 1^, and the alkaline-extractable Cr(VI) fraction increased disproportionately, from 2.92 ± 0.09 to 52.07 ± 0.11 mg kg^− 1^, so that the mobile species accounted for a progressively larger share of the total burden. Because this enrichment coincided with a shift toward alkalinity (pH 7.74 to 8.48), monitoring frameworks based on total chromium alone will understate the risk carried by these soils. Chromium loading was accompanied by measurable erosion of soil fertility organic carbon declined by up to 31.7% and exchangeable calcium and magnesium by up to 40.4%. While, irrigation water at the same sites carried elevated electrical conductivity and magnesium, resulting in constrained crop performance. Crop chromium burden was governed by bioavailability rather than by total soil loading. Tissue concentrations followed root > shoot > leaf throughout but peaked at Near-threshold rather than High Cr sites, demonstrating that the most contaminated fields are not necessarily those producing the most contaminated cane. The agronomic penalty fell on biomass rather than on sugar quality cane and stalk fresh weight declined by 25.8–31.3%, whereas sucrose, Brix, purity and fibre were largely conserved. Chromium contamination in this landscape therefore presents primarily as a tonnage constraint rather than a sugar-recovery constraint.

Bacterial communities responded as a threshold-structured rather than linearly dose-responsive ecological filter.Observed richness in High Cr fell to 74–76% than to Low Cr levels. Both richness and evenness contributed to the diversity loss. Chromium tolerance was taxonomically structured and functionally reinforced, with Actinobacterial genera enriched under exposure, a resilient species level core (*Sphingomonas panacis*, *Nitrospira lenta*) persisting across the full gradient, and predicted abundance of *azoR/acpD* and *chrA* elevated in the most contaminated stratum, together defining a candidate chromium-tolerance signature for tannery-impacted agricultural soils. Finally, principal component analysis resolved chromium loading and soil fertility onto approximately orthogonal axes, indicating that residual fertility status varies independently of contamination across these fields and identifying targeted fertility management as a hypothesis warranting experimental evaluation alongside, rather than in place of chromium remediation. Isolating the chromium tolerant lineages identified here and reintroducing them as defined consortia offers a low cost, site-specific route to decontaminating chromium affected agricultural land in this belt. Therefore, it is imperative to carry out further investigations into the potential of indigenous microbial communities for environmental remediation and to develop strategies that can enhance their functional efficiency under contaminated conditions. Such efforts would not only advance our understanding of microbial-mediated remediation processes but also contribute to the development of sustainable and site-specific approaches for mitigating environmental contamination.

## Supplementary information

Below is the link to the electronic supplementary material.

Supplementary Material 1

## Data Availability

Data will be made available upon request.

## Declarations

### Originality

The authors confirm that this manuscript is an original work and has not been published elsewhere, either in part or in full.
