# Supplementary material for: Chromium pollution and its effects on soil health, microbial diversity, and sugarcane production in the Vellore sugarcane belt: a metagenomic and geochemical assessment
Source: World J Microbiol Biotechnol. 2026 Nov 13;42(12):668. doi: 10.1007/s11274-026-05402-1 (PMC13768285; doi:10.1007/s11274-026-05402-1)
Supplement: Supplementary file 1 [file 11274_2026_5402_MOESM1_ESM.docx]

***Supplementary methods***

***S1.1 Soil and irrigation water physico-chemical properties***

Soil pH was determined in a 1:2.5 (w/v) soil to water suspension using a combined glass calomel electrode and a calibrated digital pH meter, and electrical conductivity (EC) was measured in a saturation paste extract with correction to a reference temperature (Jackson 1973). Soil organic carbon was determined by Walkley- Black wet oxidation with chromic acid and titration against ferrous sulphate and was converted to soil organic matter by multiplying by 1.724 (Walkley and Black 1934). Available nitrogen, phosphorus, potassium and sulphur and exchangeable calcium, magnesium and sodium were determined as detailed in Table 2 (Olsen et al. 1954; Subbiah and Asija 1956; Williams and Steinbergs 1959; Jackson 1973).

The pH and EC of irrigation water were measured directly with pre-calibrated electrodes. Total hardness, total alkalinity, carbonate and bicarbonate, chloride, sulphate and nitrate were determined titrimetrically or spectrophotometrically, and calcium, magnesium, sodium and potassium by versenate (EDTA) titration and flame photometry, following standard procedures (Jackson 1973; APHA 2012) and the multi parameter groundwater quality framework applied to the Walajapet region of Vellore district (Kanchana 2018; Ahmad et al. 2021; Sinduja et al. 2023).

***S1.2. Total and speciated chromium and co-occurring metals in soil and water***

Soil subsamples (0.5-1.5 g, < 150 µm) were digested with an HNO₃:HCl mixture (1:3, v/v) under microwave-assisted heating following USEPA Method 3050B, and water samples were digested for total recoverable metals following USEPA Method 3005A (Moor et al. 2001; Gaudino et al. 2007; Ahmad et al. 2021). Digests were filtered through acid washed 0.45 µm membranes, made up to 25 mL with ultrapure (Milli-Q) water, and analysed for Cr, Cd, Ni, Pb, Fe, Zn, Mn and Cu by microwave plasma atomic emission spectrometry (MP-AES; Agilent 4210, Agilent Technologies, USA), with operating conditions set according to the multi metal workflow of Ahmad et al. (2021) (Nannoni and Protano 2016; Ranieri et al. 2021). A subset of samples was reanalysed by ICP-OES for higher-sensitivity confirmation (Moor et al. 2001).

Hexavalent chromium in soil was extracted by alkaline digestion with Na_2_CO_3_/NaOH at pH > 11 (USEPA Method 3060A) to retain chromium in solution as CrO_4_^2-^; water samples were analysed directly. In both matrices Cr(VI) was quantified spectrophotometrically at 540 nm by the 1,5-diphenylcarbazide (DPC) method (Filgueiras et al. 2002; Gil-Cardeza et al. 2014; Nannoni and Protano 2016; Hamilton et al. 2018, 2020). The DPC method was adopted because of its established use in agricultural soil chromium surveys and its reproducibility over the concentration range typically encountered in tannery impacted soils of the Vellore region (Kistan et al. 2017; Hamilton et al. 2018, 2020; Sinduja et al. 2022). Trivalent chromium was obtained by difference, Cr(III) = total Cr − Cr(VI), assuming that total soil chromium is partitioned between the trivalent and hexavalent forms (Gil-Cardeza et al. 2014; Hamilton et al. 2018; Zulfiqar et al. 2023); the same relationship has been applied to Cr speciation in soils of the study region (Sinduja et al. 2022).

A reagent blank and a certified chromium reference material (NIST SRM 2701) were processed with every digestion batch, the calibration curve was verified daily, and a comparative digestion check was performed on a subset of samples as an additional quality control (Moor et al. 2001; Gaudino et al. 2007; Pueyo et al. 2001; Rauret et al. 1999).

The contamination status of each site was expressed on the chromium specific axes of total Cr, Cr(VI) and Cr(III) rather than through a generic multi metal pollution index, while Cd, Ni and Pb were retained as co-occurring analytes providing geochemical context (Kistan et al. 2017; Karthikeyan et al. 2021; Sinduja et al. 2022).

***S1.3. Chromium in sugarcane tissues***

Oven dried and ground leaf, shoot and root material (Section 2.2) was digested with an HNO_3_:HClO_4_ mixture (4:1, v/v) on a hot plate at approximately 180 °C until a clear solution was obtained (Pandey et al. 2016; Srivastava et al. 2021; Saleem et al. 2022; Zulfiqar et al. 2023). Chromium in the tissue digests was quantified by MP-AES (Agilent 4210) under the same operating conditions used for the soil and water digests; each digestion batch included a reagent blank and a plant certified reference material (Allen 1974; Mathur et al. 2016).

***S1.4. Growth, yield and juice quality***

Vegetative growth was recorded at the time of soil sampling, immediately before harvest. Cane length (cm) and root length (cm) were measured with a graduated rod and a measuring tape; cane diameter and cane girth (cm) were measured at the mid internode position with a Vernier caliper and a flexible tape, respectively; and the number of nodes per stalk and the mean internode length (cm) were recorded per stalk (Vinicius et al. 2017; Shukla et al. 2020; Saranraj et al. 2023). The length (L) and maximum width (W) of the third fully expanded leaf with ≥ 20 % green area were measured, and leaf area (AF, cm^2^) was computed by the Hermann and Câmara formula, AF = L × W × 0.75 × (N + 20), where N is the number of expanded leaves with ≥ 20 % green area (Vinicius et al. 2017; Shukla et al. 2020).

At harvest, approximately 10-12 months after planting, canes were cut at ground level from a pre marked net plot of fixed area per replicate, excluding guard rows. Total weight of stalk (kg) and single cane weight (kg) were recorded on a digital balance, and cane was crushed in a power crusher to determine the weight of extracted juice (kg), the weight of dry bagasse (kg) and juice extraction (%), calculated as the weight of extracted juice expressed as a percentage of the weight of cane crushed (Karpagam et al. 2019; Meena et al. 2020; Shukla et al. 2020; Saranraj et al. 2023). In the extracted juice, brix (%) was measured with a hand refractometer, sucrose (pol %) was determined at 589 nm by ICUMSA Sucromat polarimetry, and fibre content (%) was determined by rapipol extraction. Juice purity (%) was calculated as the ratio of sucrose to brix, and commercial cane sugar (CCS, %) was computed following the recommendations of the International Society of Sugar Cane Technologists (Karpagam et al. 2019; Shukla et al. 2020).

Table S1. Irrigation water quality parameters across chromium-contamination categories. Percentage change relative to Low. Superscript letters denote Tukey HSD post-hoc groupings (α = 0.05) within each row; categories sharing a letter are not significantly different from one another.

| Parameter | Low threshold limit | Near threshold limit | High threshold limit |
| --- | --- | --- | --- |
| pH | 7.94 ± 0.25 ^a^ | 7.85 ± 0.06 ^a^ (−1.2%) | 7.91 ± 0.04 ^a^ (−0.4%) |
| Ec (ds m-1) | 3.26 ± 0.01 ^c^ | 5.29 ± 0.01 ^b^ (+62.1%) | 5.90 ± 0.09 ^a^ (+80.8%) |
| Chloride (mg/L) | 854.48 ± 5.81 ^a^ | 668.48 ± 1.36 ^b^ (−21.8%) | 662.54 ± 4.96 ^b^ (−22.5%) |
| Sulphate (mg/L) | 82.33 ± 0.45 ^a^ | 73.57 ± 0.70 ^b^ (−10.6%) | 67.03 ± 0.02 ^c^ (−18.6%) |
| Carbonate (mg/L) | 68.33 ± 0.02 ^b^ | 94.67 ± 1.09 ^a^ (+38.5%) | 92.67 ± 0.03 ^a^ (+35.6%) |
| bicarbonate (mg/L) | 213.33 ± 4.72 ^b^ | 204.33 ± 1.88 ^b^ (−4.2%) | 231.33 ± 2.05 ^a^ (+8.4%) |
| Calcium (mg/L) | 169.00 ± 3.91 ^b^ | 244.00 ± 3.07 ^a^ (+44.4%) | 232.67 ± 2.69 ^a^ (+37.7%) |
| Magnesium (mg/L) | 86.00 ± 0.67 ^c^ | 188.00 ± 1.15 ^b^ (+118.6%) | 248.33 ± 0.51 ^a^ (+188.8%) |
| Potassium (mg/L) | 7.30 ± 0.23 ^b^ | 9.63 ± 0.03 ^a^ (+32.0%) | 7.10 ± 0.01 ^b^ (−2.7%) |

Table S2. Leaf, shoot and root tissue values across soil chromium-contamination categories. Percentage change relative to Low. Superscript letters denote Tukey HSD post-hoc groupings (α = 0.05) within each row; categories sharing a letter are not significantly different from one another.

| Parameter | Low threshold limit | Near threshold limit | High threshold limit |
| --- | --- | --- | --- |
| Leaf | 0.87 ± 0.01 ^c^ | 2.13 ± 0.00 ^a^ (+146.2%) | 1.33 ± 0.00 ^b^ (+53.8%) |
| shoot | 1.33 ± 0.02 ^c^ | 5.03 ± 0.00 ^a^ (+277.5%) | 2.57 ± 0.03 ^b^ (+92.5%) |
| root | 1.87 ± 0.05 ^c^ | 8.03 ± 0.06 ^a^ (+330.4%) | 4.40 ± 0.05 ^b^ (+135.7%) |

Table S3. Vegetative growth parameters of sugarcane across soil chromium-contamination categories. Percentage change relative to Low. Superscript letters denote Tukey HSD post-hoc groupings (α = 0.05) within each row; categories sharing a letter are not significantly different from one another.

| Parameter | Low threshold limit | Near threshold limit | High threshold limit |
| --- | --- | --- | --- |
| Cane length (cm) | 202.17 ± 1.93 ^a^ | 197.83 ± 2.83 ^a^ (−2.1%) | 146.33 ± 2.29 ^b^ (−27.6%) |
| Root Length (cm) | 42.43 ± 0.14 ^a^ | 41.13 ± 0.21 ^b^ (−3.1%) | 37.23 ± 0.20 ^c^ (−12.3%) |
| Leaf length (cm) | 149.87 ± 0.20 ^a^ | 151.67 ± 0.72 ^a^ (+1.2%) | 142.50 ± 2.28 ^b^ (−4.9%) |
| Leaf Width (cm) | 5.65 ± 0.01 ^a^ | 5.41 ± 0.03 ^b^ (−4.2%) | 4.95 ± 0.05 ^c^ (−12.4%) |
| Leaf area (Cm2) | 634.78 ± 4.10 ^a^ | 615.89 ± 5.66 ^a^ (−3.0%) | 530.59 ± 7.04 ^b^ (−16.4%) |
| Cane diameter (cm) | 2.82 ± 0.04 ^a^ | 2.51 ± 0.01 ^b^ (−10.9%) | 2.19 ± 0.02 ^c^ (−22.5%) |
| Cane girth (cm) | 8.87 ± 0.04 ^a^ | 7.90 ± 0.05 ^b^ (−10.9%) | 6.87 ± 0.04 ^c^ (−22.6%) |
| Number of nodes | 21.00 ± 0.51 ^b^ | 24.33 ± 0.40 ^a^ (+15.9%) | 18.00 ± 0.17 ^c^ (−14.3%) |
| Internode length (cm) | 10.23 ± 0.15 ^b^ | 11.23 ± 0.03 ^a^ (+9.8%) | 8.80 ± 0.12 ^c^ (−14.0%) |

Table S4. Cane yield and juice-quality attributes across soil chromium-contamination categories. Percentage change relative to Low. Superscript letters denote Tukey HSD post-hoc groupings (α = 0.05) within each row; categories sharing a letter are not significantly different from one another

| Parameter | Low threshold limit | Near threshold limit | High threshold limit |
| --- | --- | --- | --- |
| Total weight of stalk | 3.55 ± 0.10 ^a^ | 3.44 ± 0.05 ^a^ (−3.1%) | 2.63 ± 0.01 ^b^ (−25.8%) |
| Single cane weight (Kg) | 1.18 ± 0.00 ^a^ | 0.69 ± 0.01 ^c^ (−41.9%) | 0.81 ± 0.01 ^b^ (−31.3%) |
| Sucrose (%) or Pol % 589nm | 19.23 ± 0.18 ^a^ | 17.30 ± 0.29 ^b^ (−10.0%) | 18.46 ± 0.29 ^a^ (−4.0%) |
| Brix % | 20.10 ± 0.49 ^a^ | 18.90 ± 0.04 ^a^ (−6.0%) | 19.33 ± 0.24 ^a^ (−3.8%) |
| Purity % | 95.68 ± 2.60 ^a^ | 91.96 ± 0.16 ^a^ (−3.9%) | 95.35 ± 1.46 ^a^ (−0.3%) |
| CCS % (Commercial Cane Sugar) | 13.97 ± 0.20 ^a^ | 12.33 ± 0.00 ^c^ (−11.7%) | 13.40 ± 0.09 ^b^ (−4.0%) |
| Weight of the dry bagasse | 1.06 ± 0.04 ^a^ | 0.97 ± 0.01 ^a^ (−7.8%) | 0.73 ± 0.01 ^b^ (−30.9%) |
| Fibre content (%) | 29.58 ± 0.91 ^a^ | 28.47 ± 0.37 ^a^ (−3.8%) | 27.67 ± 0.00 ^a^ (−6.5%) |
| Weight of Extracted Juice | 2.50 ± 0.05 ^a^ | 2.47 ± 0.02 ^a^ (−1.0%) | 1.91 ± 0.01 ^b^ (−23.7%) |
| Juice extraction (%) | 70.42 ± 2.25 ^a^ | 71.66 ± 0.15 ^a^ (+1.8%) | 72.33 ± 0.12 ^a^ (+2.7%) |

**Supplementary Figures**

**Fig. S1.** Irrigation Water physicochemical parameters across the chromium gradient.

**Fig. S2.** Chromium accumulation in sugarcane plant tissues.

**Fig. S3.** Sugarcane vegetative growth parameters. Mean cane length, root length, leaf length, leaf width, and leaf area across the chromium gradient, with percentage change relative to Low.

**Fig. S4.** Sugarcane stalk morphometric parameters. Mean cane diameter, cane girth, internode length, and number of nodes across the chromium gradient, with percentage change relative to Low.

**Fig. S5.** Juice quality and cane yield parameters. Mean sucrose (Pol%), Brix%, purity%, CCS%, total stalk weight, and single cane weight across the chromium gradient.

**Fig. S6.** Bagasse and juice extraction parameters. Mean weight of dry bagasse, fibre content, weight of extracted juice, and juice extraction efficiency across the chromium gradient.

**Fig. S7.** Genus level co-occurrence network. Spearman correlation network among the most abundant bacterial genera, arranged circularly with node size proportional to mean relative abundance. Red edges denote positive correlations, and blue edges denote negative correlations between genus pairs.
